# GDSL lipases modulate immunity through lipid homeostasis in rice

**DOI:** 10.1371/journal.ppat.1006724

**Published:** 2017-11-13

**Authors:** Mingjun Gao, Xin Yin, Weibing Yang, Sin Man Lam, Xiaohong Tong, Jiyun Liu, Xin Wang, Qun Li, Guanghou Shui, Zuhua He

**Affiliations:** 1 National Key Laboratory of Plant Molecular Genetics, CAS Center for Excellence in Molecular Plant Sciences/Institute of Plant Physiology & Ecology, Chinese Academy of Sciences, Shanghai, China; 2 State Key Laboratory of Molecular Developmental Biology, Institute of Genetics and Developmental Biology, Chinese Academy of Sciences, Beijing, China; 3 China National Rice Research Institute, Hangzhou, China; The Ohio State University, UNITED STATES

## Abstract

Lipids and lipid metabolites play important roles in plant-microbe interactions. Despite the extensive studies of lipases in lipid homeostasis and seed oil biosynthesis, the involvement of lipases in plant immunity remains largely unknown. In particular, GDSL esterases/lipases, characterized by the conserved GDSL motif, are a subfamily of lipolytic enzymes with broad substrate specificity. Here, we functionally identified two GDSL lipases, OsGLIP1 and OsGLIP2, in rice immune responses. Expression of *OsGLIP1* and *OsGLIP2* was suppressed by pathogen infection and salicylic acid (SA) treatment. *OsGLIP1* was mainly expressed in leaf and leaf sheath, while *OsGLIP2* showed high expression in elongating internodes. Biochemical assay demonstrated that OsGLIP1 and OsGLIP2 are functional lipases that could hydrolyze lipid substrates. Simultaneous down-regulation of *OsGLIP1* and *OsGLIP2* increased plant resistance to both bacterial and fungal pathogens, whereas disease resistance in *OsGLIP1* and *OsGLIP2* overexpression plants was significantly compromised, suggesting that both genes act as negative regulators of disease resistance. OsGLIP1 and OsGLIP2 proteins mainly localize to lipid droplets and the endoplasmic reticulum (ER) membrane. The proper cellular localization of OsGLIP proteins is indispensable for their functions in immunity. Comprehensive lipid profiling analysis indicated that the alteration of *OsGLIP* gene expression was associated with substantial changes of the levels of lipid species including monogalactosyldiacylglycerol (MGDG) and digalactosyldiacylglycerol (DGDG). We show that MGDG and DGDG feeding could attenuate disease resistance. Taken together, our study indicates that OsGLIP1 and OsGLIP2 negatively regulate rice defense by modulating lipid metabolism, thus providing new insights into the function of lipids in plant immunity.

## Author summary

Lipases are a large family of enzymes conferring lipid metabolism. Lipids and their metabolites play diverse roles in plant growth as well as response to environmental stimuli. Accumulating evidence implicates lipids as signaling molecules mediating plant immunity. Therefore, lipases are presumed to be actively involved in plant defense responses. Based on gene expression profiling, we have identified two functional GDSL lipases, encoded by *OsGLIP1* and *OsGLIP2*, whose expression was suppressed by pathogen infection in the model cereal rice. Both OsGLIP1 and OsGLIP2 proteins localize to lipid droplets and the endoplasmic reticulum (ER) membrane, and they likely coordinate lipid metabolism with differential but complementary expression patterns in tissues and developmental stages. Consequently, alteration of *OsGLIP* gene expression was associated with substantial changes of lipid abundance and plant disease resistance. Our work identifies and characterizes two lipases that function as negative regulators of plant immune responses, strengthening the understanding of lipid metabolism in plant-microbe interactions.

## Introduction

Plants are continuously challenged by invading microorganisms, some of which are pathogens that threaten plant survival and cause a big loss of production in crops. During evolution, multiple layers of defense systems have been adopted by plant hosts in the fight against pathogen attack, such as physical barriers including cell wall and epidermal cuticles [1]. More importantly, plants have developed two elaborate immune systems, pathogen-associated molecular pattern (PAMP)-triggered immunity (PTI) and effector-triggered immunity (ETI). Perception of PAMPs conserved among different microorganisms by plant pattern recognition receptors (PRRs) triggers PTI, which constitutes the basal layer of plant defense. ETI is specifically induced when pathogen secreted effectors are recognized by host resistance (R) proteins, often leading to hypersensitive response (HR), which is a more robust defense response usually associated with reactive oxygen species (ROS) burst and programmed cell death [2].

Accumulating evidence has suggested lipids as important regulators of plant defense [3,4]. Cutin, a polymer matrix composed of short-chain fatty acids (FA), and wax, a mixture of very-long-chain fatty acids, form a physical barrier preventing pathogen infection [5]. Lipids and their derivatives have also been implicated as signaling molecules that could modulate plant immunity [6]. For instance, the well-known plant defense hormone, jasmonic acid (JA), is a lipid-derived molecule [7]. Oleic acid (depicted as 18:1), an endogenous unsaturated fatty acid, has been shown to suppress defense response [8,9]. Recent studies have also emphasized the important roles of lipids in the induction of systemic acquired resistance (SAR), a defense mechanism occurring in the systemic organs to protect plants against subsequent attack [10,11]. The identification of *DIR1* (*DEFECTIVE IN INDUCED RESISTANCE 1*) as a potential lipid transfer protein provided strong evidence that a lipid-derived molecule was involved in long distance defense signaling [12]. Further studies characterized azelaic acid (AzA), a C9 dicarboxylic acid derived from C18 FAs and glycerol-3-phosphate (G3P, the precursor for all plant glycerolipids), as critical inducers of plant SAR [13,14]. G3P and AzA cooperate with DIR1 and another predicted lipid transfer protein AZELAIC ACID INDUCED 1 (AZI1) to form a feedback regulatory loop that regulates systemic immunity in plants [15]. Further investigation of DIR1 and AZI1 lipid transfer activities and their target lipid molecules would provide invaluable insights into the roles of lipids in plant immunity.

Lipid metabolism is mainly catalyzed by lipases. GDSL lipases are a newly discovered subclass of lipolytic enzymes characterized by a GDSL motif and are further classified as SGNH hydrolase due to the highly conserved residues Ser-Gly-Asn-His [16]. Despite the extensive studies in bacteria, relatively little is known about plant GDSL lipases although they have been identified in various plant species [3]. Interestingly, the Arabidopsis GDSL lipase AtGLIP1 has been shown to possess anti-microbial activity and modulates resistance to *Alternaria brassicicola* in association with ethylene signaling [17,18], while its closest homolog AtGLIP2 plays a role in defense by suppressing auxin response [19]. In hot pepper, CaGLIP1 is involved in pathogen and wound defense [20]. Moreover, TcGLIP from *Tanacetum cinerariifolium* confers acyltransferase activity responsible for the biosynthesis of natural insecticide pyrethrin [21].

Rice (*Oryza sativa* L.) serves as the major food crop supporting nearly half of the world population. The production and quality of rice are severely challenged by a variety of pathogens. Bacterial blight caused by *Xanthomonas oryzae* pv. *oryzae* (*Xoo*) and rice blast caused by *Magnaporthe oryzae* (*M*. *oryzae*) are major diseases that affect rice production and quality. Bioinformatics analysis identified a large family of GDSL lipases (~114 members) in the rice genome, but little is known about their functions [22,23]. Here we report the identification and functional characterization of two GDSL lipases, OsGLIP1 and OsGLIP2, and we show that the two lipases are involved in rice disease resistance. With comprehensive genetic, biochemical, and lipid profiling assays, our work indicated that OsGLIP1 and OsGLIP2 are functional lipases involved in lipid metabolism and negatively modulate rice immune responses.

## Results

### Characterization of two functional lipases *OsGLIP1* and *OsGLIP2* in rice

**Fig 1:**
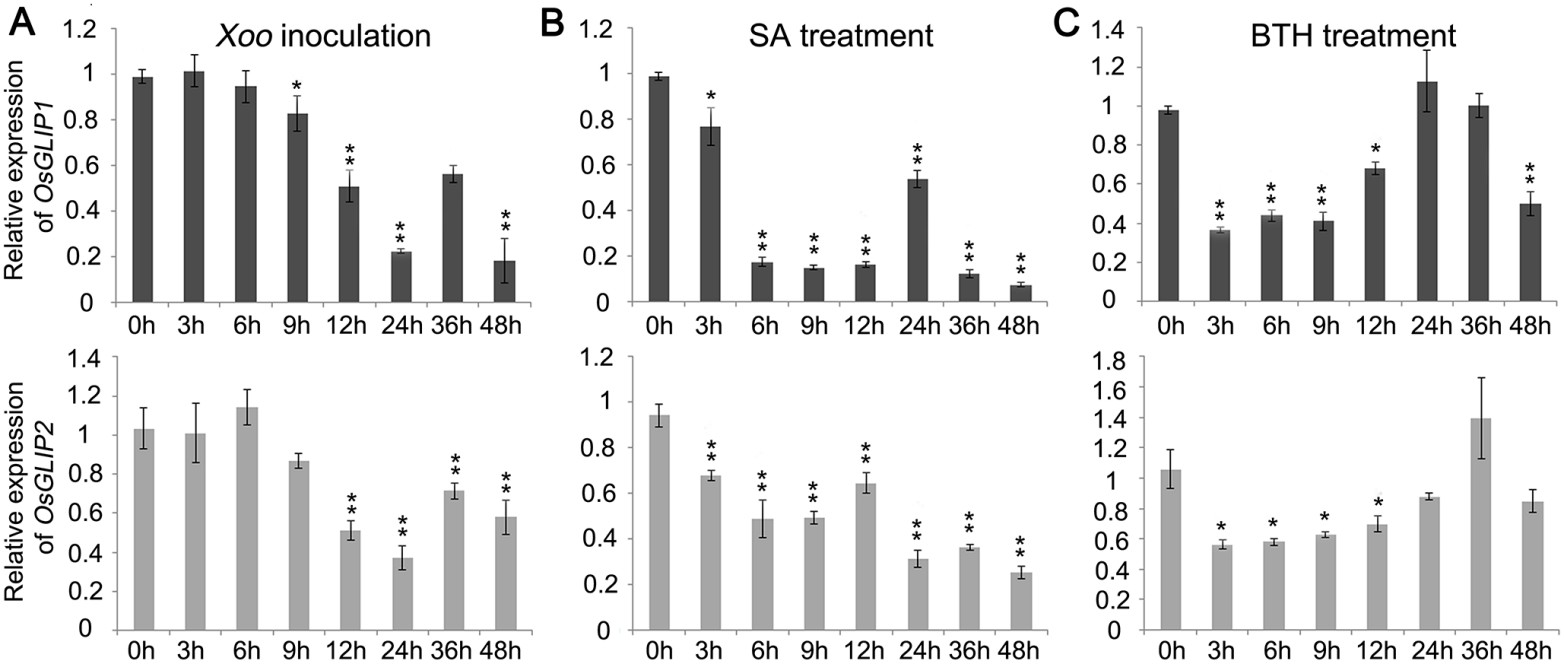
Expression of *OsGLIP1* and *OsGLIP2* was suppressed in responses to pathogen infection and chemical treatments. (A) Down-regulation of *OsGLIP1* and *OsGLIP2* expression in eight-week-old plants infected with *Xoo* (strain PXO99A) in a time course of 48 hours. (B, C) Down-regulation of *OsGLIP1* and *OsGLIP2* expression in two-week-old seedlings sprayed with 1 mM SA (B) or 300 μM BTH (C). All treatments were repeated for three times with similar results (A-C). The rice *Actin1* gene was used as an internal control. Data are shown as means ± SD from three biological replicates (Student’s *t*-test, *P < 0.05, **P < 0.01).

In order to identify new components involved in rice disease resistance, we had previously performed whole genome transcriptome analysis of rice genes in responses to *Xoo* and *M*. *oryzae* infection [24]. We identified a GDSL lipase gene, *Os06g0129600* (*OsGLIP1*), the expression of which was suppressed after *Xoo* inoculation (fold change = 3.3). The down-regulation of *OsGLIP1* by *Xoo* was confirmed by qRT-PCR, which was down-regulated and decreased to the lowest level at 24h post-inoculation (hpi) (Fig 1A). The suppression of gene expression following pathogen infection was also observed for the closest homolog of *OsGLIP1*, *Os06g0156700*, which we designated as *O*s*GLIP2* (Fig 1A). Since SA is the major defense signaling molecule, we further examined the expression of *OsGLIP1* and *OsGLIP2* after SA treatment. The amount of *OsGLIP1* and *OsGLIP2* transcripts started to decrease as soon as 3h after SA treatment and reached to the lowest level in 6h to 9h, causing a faster expression inhibition in comparison with pathogen inoculation (Fig 1B). Similarly, treatment with BTH, a functional analog of SA, resulted in similar inhibition of *OsGLIP1* and *OsGLIP2* expression (Fig 1C). The results suggest that *OsGLIP1* and *OsGLIP2* might function in rice immune responses.

**Fig 2:**
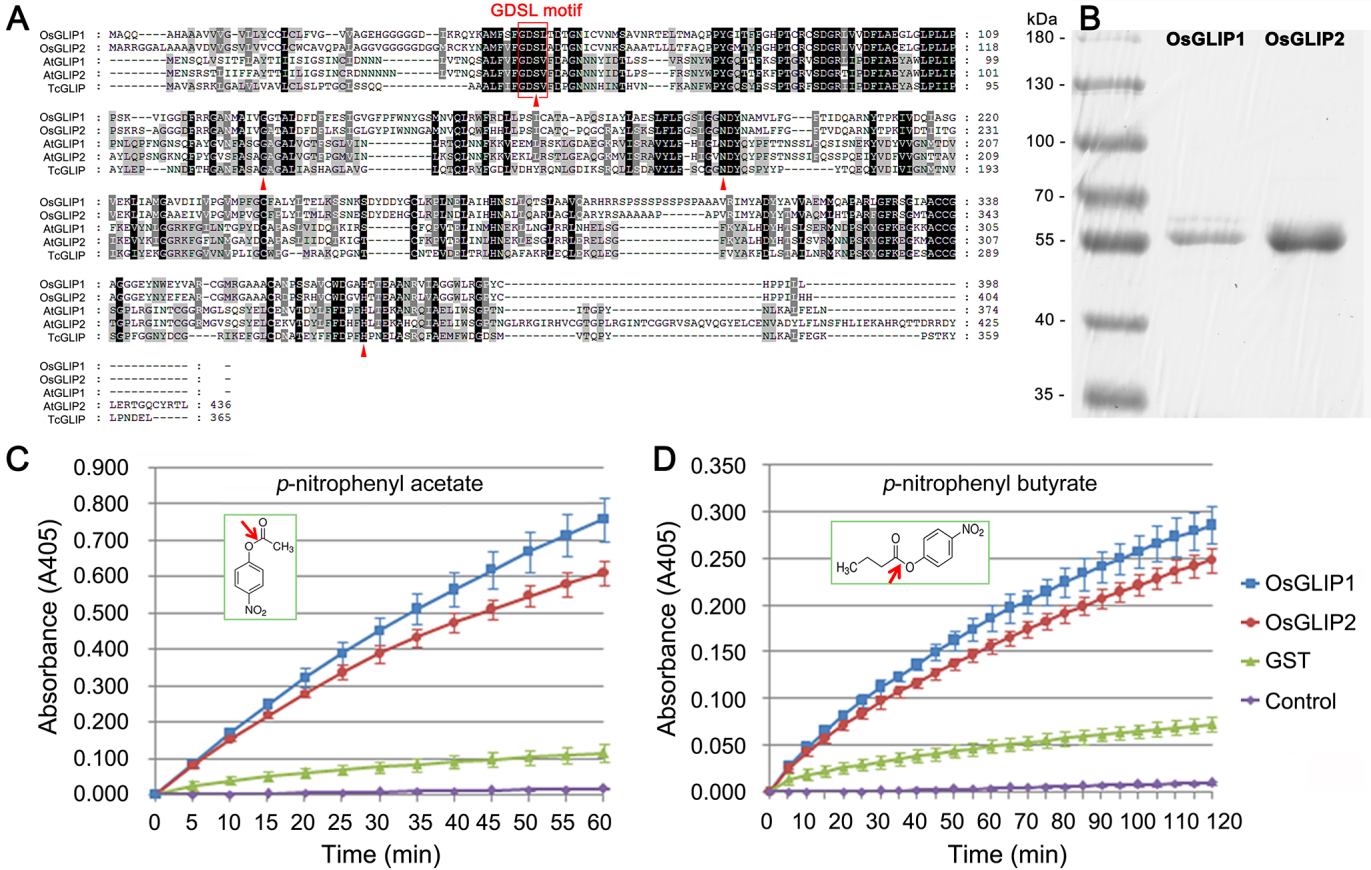
OsGLIP1 and OsGLIP2 exhibit lipase activities. (A) Alignment of OsGLIP1 and OsGLIP2 amino acid sequences with functionally known homologues from *Arabidopsis* and *Tanacetum cinerariifolium*. Sequences were aligned using Genedoc. (B) Expression and purification of recombinant OsGLIP1-GST and OsGLIP2-GST proteins in *E*. *coli*. (C, D) Lipase activities of OsGLIP1 and OsGLIP2. OsGLIP1 and OsGLIP2 were incubated with *p*-nitrophenyl acetate (C) and *p*-nitrophenyl butyrate (D) at 30°C. The absorbance readings were collected every 5 minutes in a time course of 60 min or 120 min. The substrates were incubated with either GST or no protein as controls. Data are shown as means ± SD (*n* = 3).

The highly conserved amino acid sequences of GDSL lipases among different organisms indicate that they might exhibit similar catalytic activities [25]. Phylogenetic analysis of rice GDSL lipases revealed that OsGLIP1 and OsGLIP2 group into the same clade, with 73% identity in amino acid sequences (S1 Fig). Further comparison of the OsGLIP1 and OsGLIP2 protein sequences with orthologues from other plant species revealed a high degree of similarity, especially for the GDSL/V motif in the N-terminal and the four invariant key catalytic residues Ser, Gly, Asn and His in the functional blocks (Fig 2A). To test the lipase activity of OsGLIP1 and OsGLIP2, we attempted to express GST-tagged OsGLIP1 and OsGLIP2 recombinant proteins in *E*. *coli*. However, we failed to express the full-length fusion proteins probably due to the signal peptides as predicted by SignalP (http://www.cbs.dtu.dk/services/SignalP) [26]. Instead, truncated OsGLIP proteins without the signal peptides (OsGLIP1^Δ29^ and OsGLIP2^Δ35^) could be produced successfully (Fig 2B). The recombinant proteins were then purified and incubated with *p*-nitrophenyl acetate and *p*-nitrophenyl butyrate, two synthetic substrates generally used for lipase activity assay [17,19]. Compared to the empty control and GST alone, both OsGLIP1^Δ29^ and OsGLIP2^Δ35^ hydrolyzed the lipid substrates with high efficiency (Fig 2C and 2D), demonstrating that OsGLIP1 and OsGLIP2 are functional lipases.

### OsGLIP1 and OsGLIP2 modulate plant lipid homeostasis

To further investigate the roles of OsGLIP1 and OsGLIP2 in lipid metabolism in plants, we performed lipid profiling assay in transgenic plants with altered *OsGLIP1* and *OsGLIP2* expression. The maize *Ubiquitin1* promoter (*Ubi1*) was used to drive *OsGLIP1* and *OsGLIP2* overexpression in wild-type plants. For reduced expression, a conserved fragment from the *OsGLIP1* and *OsGLIP2* coding regions was selected to generate an RNA interference (RNAi) construct that targets both genes simultaneously. qRT-PCR analysis revealed that the transcript levels of both genes was increased or decreased in independent transgenic lines (S2A and S2B Fig).

**Fig 3:**
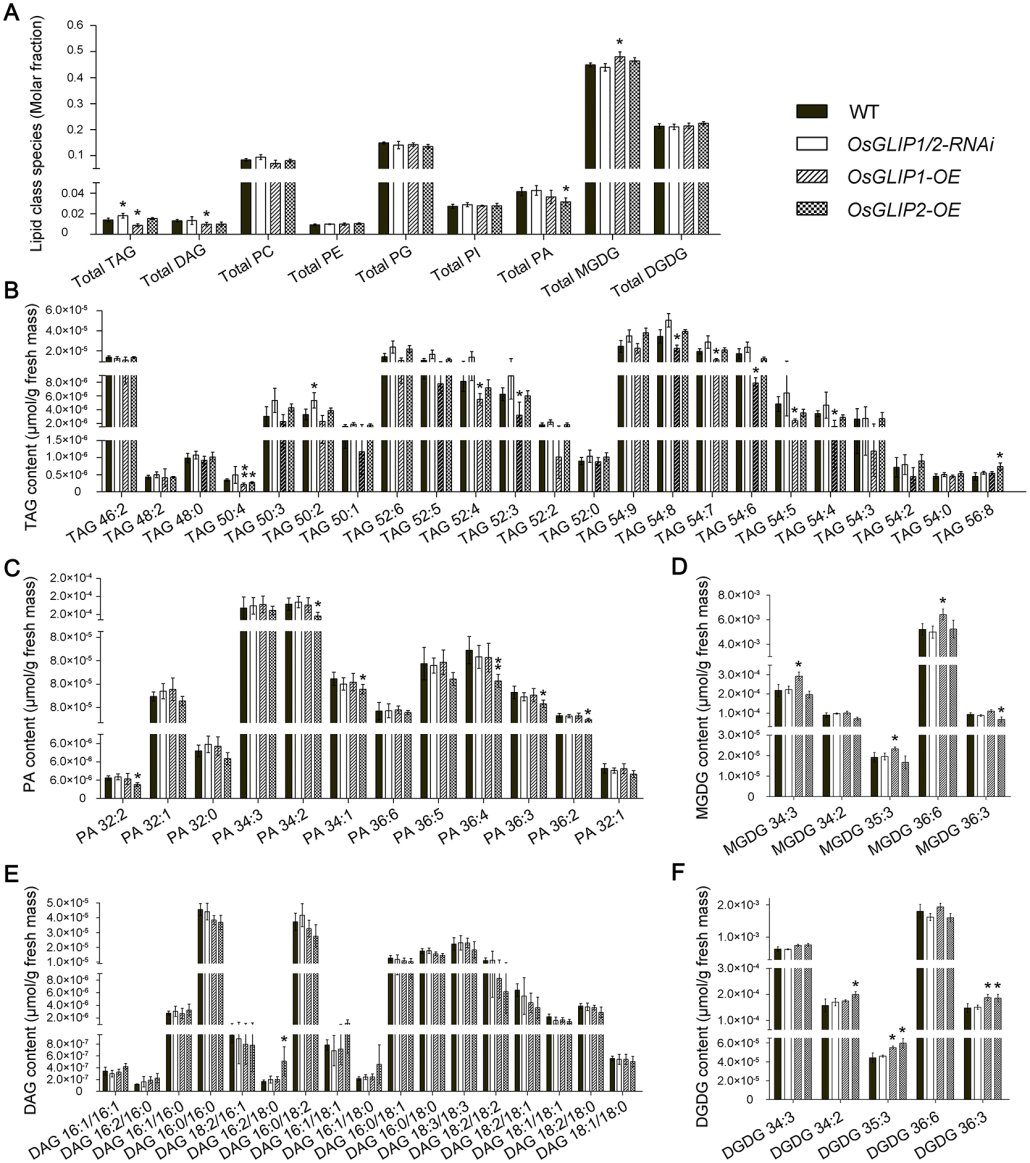
Lipidomic profiling of *OsGLIP1/2-RNAi* and overexpression plants. Leaves from six individual plants (eight-week-old) were mixed as one sample from three representative transgenic lines of each transgene were used to normalize samples. Five leaf samples each genetic background were statistically analysed. (A) Total lipid composition in leaves of eight-week-old plants. (B-F) Abundance of individual lipid species, TAG (B), PA (C), MGDG (D), DAG (E) and DGDG (F). The lipid structures are presented as the number of carbon atoms: total double bonds in the fatty acyl groups. Data are shown as means ± SD (*n* = 5) of mixed leaf samples from three representative transgenic lines. *P < 0.05 or **P < 0.01, by Student’s *t*-test and Bonferroni correction for multiple (three comparisons) tests.

Lipids were extracted from the leaves of grouped *OsGLIP1* and *OsGLIP2* overexpression and silencing plants and analysed by reverse phase high-performance liquid chromatography/electrospray ionization tandem mass spectrometry (RP-HPLC/ESI/MS/MS). As shown in Fig 3A, although no significant difference was detected for the amount of phospholipids PC (phosphatidylcholine), PE (phosphatidylethanolamine), PG (phosphatidylglycerol) and PI (phosphatidylinositol) in the RNAi and overexpression transgenic plants, the abundance of other lipids were substantially changed. For instance, total TAG was significantly increased in *OsGLIP1/2-RNAi* and decreased in *OsGLIP1-OE* plants. By contrast, total MGDG levels increased in *OsGLIP1* overexpression plants. Enhanced expression of *OsGLIP1* also led to reduction in DAG levels while *OsGLIP2* overexpression decreased PA levels. We further compared the individual lipid molecular species (Fig 3B–3F and S3 Fig), showing that TAG molecules such as TAG50, TAG52 and TAG54 containing polyunsaturated FAs either markedly increased in *OsGLIP1/2-RNAi* plants or decreased in *OsGLIP1-OE* plants, respectively (Fig 3B). While in *OsGLIP2-OE* plants, many PA species decreased significantly (Fig 3C), and some TAG species accumulated (Fig 3B). We also observed significant increase in levels of MGDG and DGDG molecules in *OsGLIP1-OE* and *OsGLIP2-OE* plants (Fig 3D and 3F). These results, taken together, suggest that OsGLIP1 and OsGLIP2 function in lipid metabolism *in planta* and these two lipases exhibit overlapping as well as distinct properties in lipid metabolism.

### *OsGLIP1* and *OsGLIP2* affect rice resistance to bacterial pathogen

**Fig 4:**
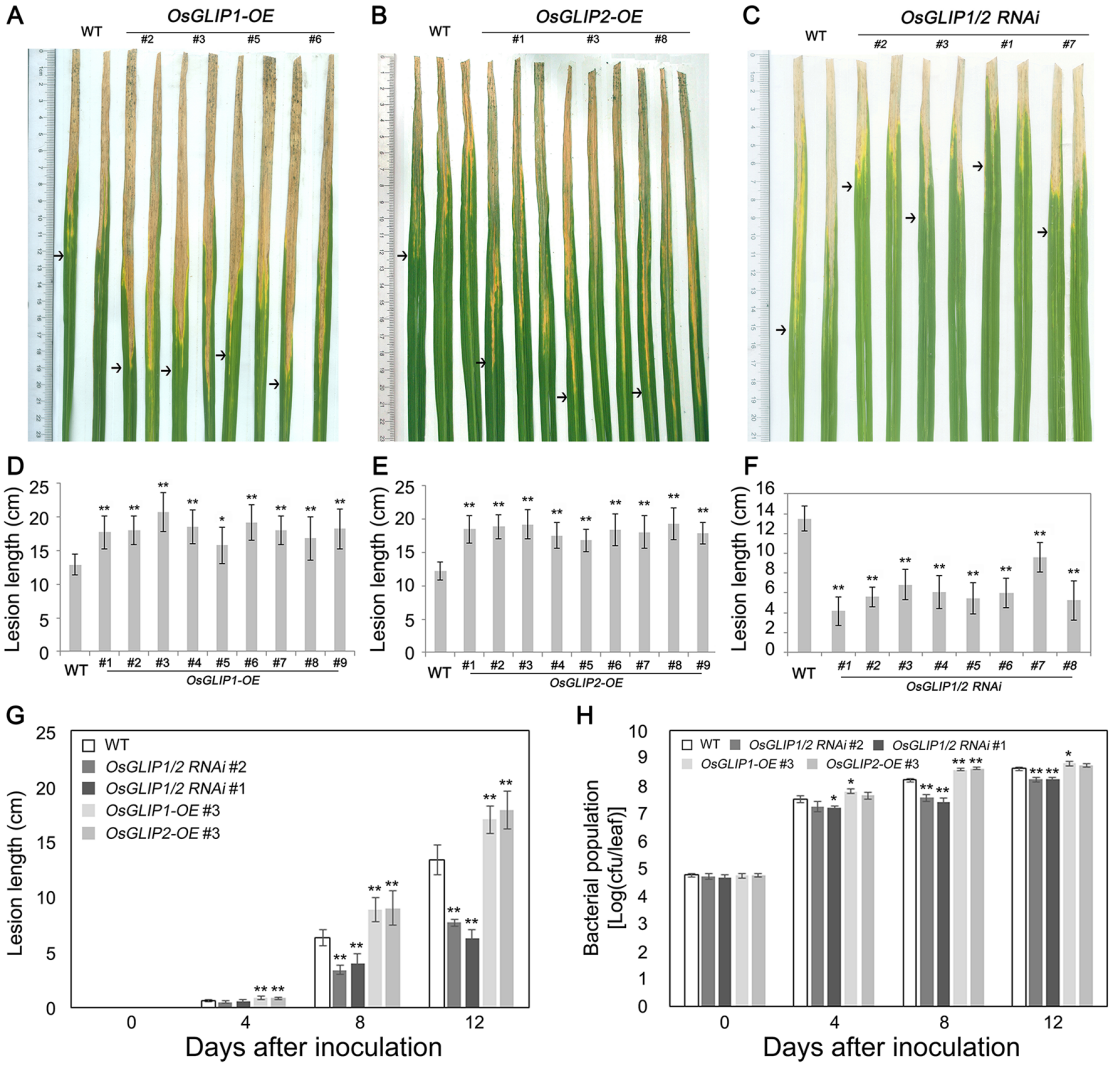
Disease resistance to bacterial blight in *OsGLIP1-OE*, *OsGLIP2-OE* and *OsGLIP1/2*-*RNAi* plants. (A-F) Lesions and statistical analysis of lesion lengths of representative *OsGLIP1-OE* (A and D), *OsGLIP2-OE* (B and E) and *OsGLIP1/2*-*RNAi* (C and F) lines (eight-week-old) inoculated with bacterial pathogen *Xoo* at 14 dpi, with the wild type (TP309, WT) as control. Arrows indicate the bottoms of lesions. Data are shown as means ± SD (*n* > 10). Asterisks indicate significant difference in comparison with the wild-type control (Student’s *t*-test, *P < 0.05; ** *P* < 0.01). (G) Disease development during 12 days of inoculation in the representative lines of *OsGLIP1-OE*, *OsGLIP2-OE* and *OsGLIP1/2*-*RNAi*, compared with the wild type. Data are shown as means ± SD (*n* > 10). Asterisks indicate significant difference in comparison with the wild-type control (Student’s *t*-test, ** *P* < 0.01). (H) Bacterial growth during 12 days of inoculation in the representative lines of *OsGLIP1-OE*, *OsGLIP2-OE* and *OsGLIP1/2*-*RNAi*, compared with the wild type. Data are shown as means ± SD (*n* = 3). Asterisks indicate significant difference in comparison with the wild-type control (Student’s *t*-test, *P < 0.05; ** *P* < 0.01).

Due to the important roles of lipids in plant immunity [3,4,6], we postulated that OsGLIP1 and OsGLIP2 might respond to pathogen infection and modulate lipid homeostasis to regulate rice immune responses. To explore the roles of OsGLIP1 and OsGLIP2 in rice immunity, *OsGLIP1* and *OsGLIP2* overexpression and RNAi plants were infected with bacterial blight pathogen *Xoo* (Fig 4A–4C). Compared to the wild type, the lesion was significantly longer in *OsGLIP1-OE* and *OsGLIP2-OE* but shorter in *OsGLIP1/2*-*RNAi* plants (Fig 4D–4F). We then chose representative lines from *OsGLIP1-OE*, *OsGLIP2-OE* and *OsGLIP1/2*-*RNAi* transgenic plants to further analyze the bacterial lesion development and growth in leaves after infection. The results showed that disease lesions expanded more quickly and bacteria grew faster in both *OsGLIP1* and *OsGLIP2* overexpression plants; while reduction of *OsGLIP1* and *OsGLIP2* expression significantly inhibited *Xoo* growth in comparison with the wild-type control, indicating marked increase of resistance against bacterial blight (Fig 4G and 4H). Taken together, pathogen inoculation assays indicated that *OsGLIP1* and *OsGLIP2* play a negative role in rice defense.

**Fig 5:**
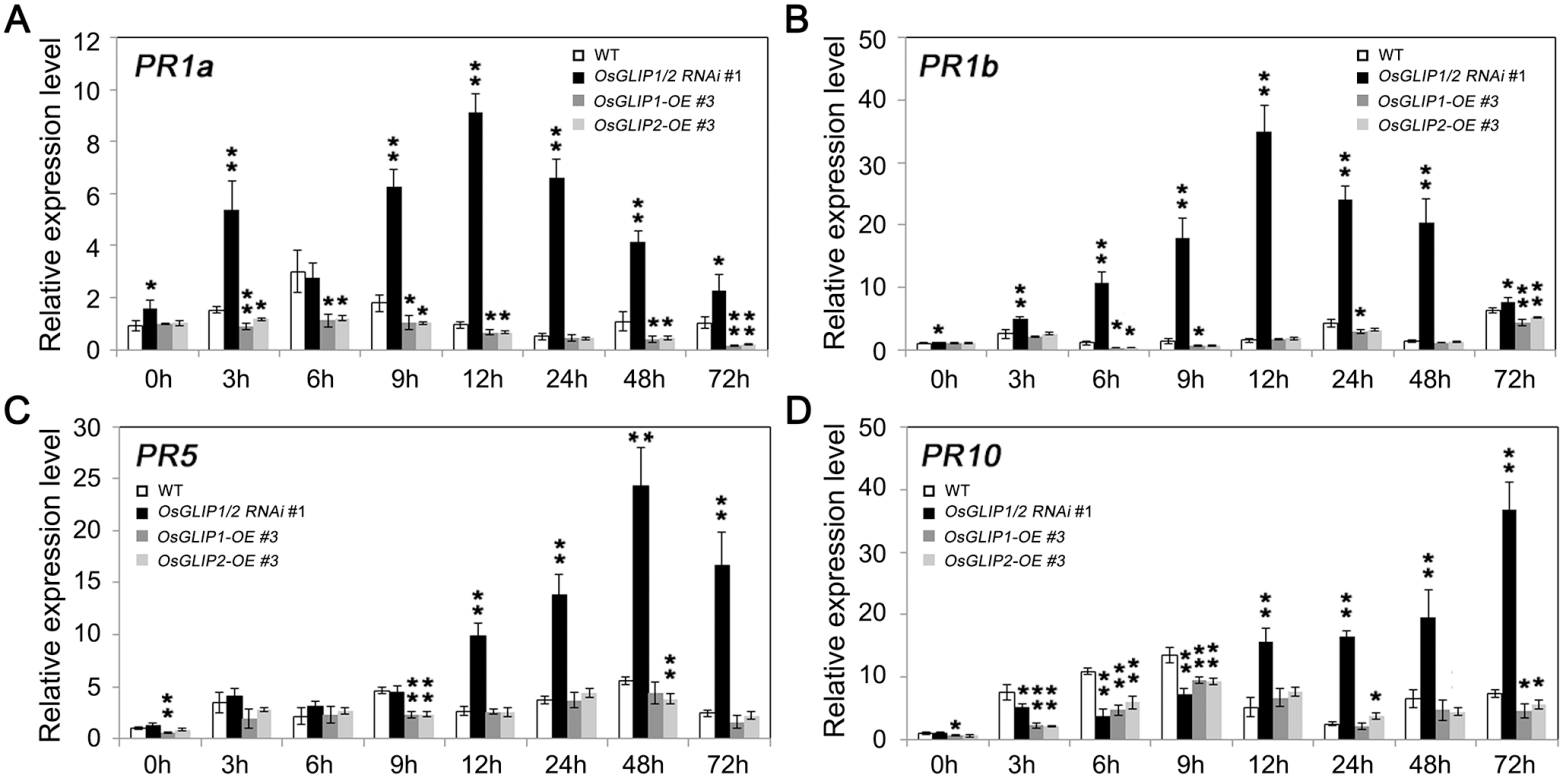
Altered expression of pathogenesis-related (*PR*) genes in *OsGLIP1* transgenic plants. Eight-week-old transgenic and wild-type plants were inoculated with *Xoo* (strain PXO99A). The induction of the *PR* genes, *PR1a* (A), *PR1b* (B), *PR5* (C) and *PR10* (D), in response to pathogen infection was compromised in *OsGLIP1-OE* plants, while silencing of both *OsGLIP1* and *OsGLIP2* significantly promoted the induction of the *PR* genes. Data shown are means ± SD from three biological replicates. Asterisks indicate significant difference in comparison with the wild-type control (Student’s *t*-test, *P < 0.05, **P < 0.01).

Activation of plant defense responses after pathogen infection is accompanied by up-regulation of pathogenesis-related (*PR*) genes [27]. We monitored the expression of *PR* genes in response to pathogen attack in the *OsGLIP1* and *OsGLIP2* transgenic lines. The leaves of transgenic as well as wild-type plants were inoculated with *Xoo* and samples were collected at different time points. We observed that the expression of *PR1a*, *PR1b*, *PR5* and *PR10* were usually induced after *Xoo* inoculation in the wild-type plants. Consistent with the enhanced disease resistance, *PR* genes showed much higher induction in *OsGLIP1/2-RNAi* plants, whereas their induction was attenuated in the *OsGLIP1-OE* and *OsGLIP2-OE* overexpression plants (Fig 5A–5D). It has been shown that *PR1a* and *PR1b* are the marker genes indicative of SA signaling activation while *PR5* and *PR10* are involved in both SA and JA signaling [28,29], the altered expression of the *PR* genes implied that the *OsGLIP* genes might affect rice immunity through indirectly modifying the defense hormone signaling pathways.

### *OsGLIP1* and *OsGLIP2* affect rice resistance to fungal pathogen *M*. *oryzae*

**Fig 6:**
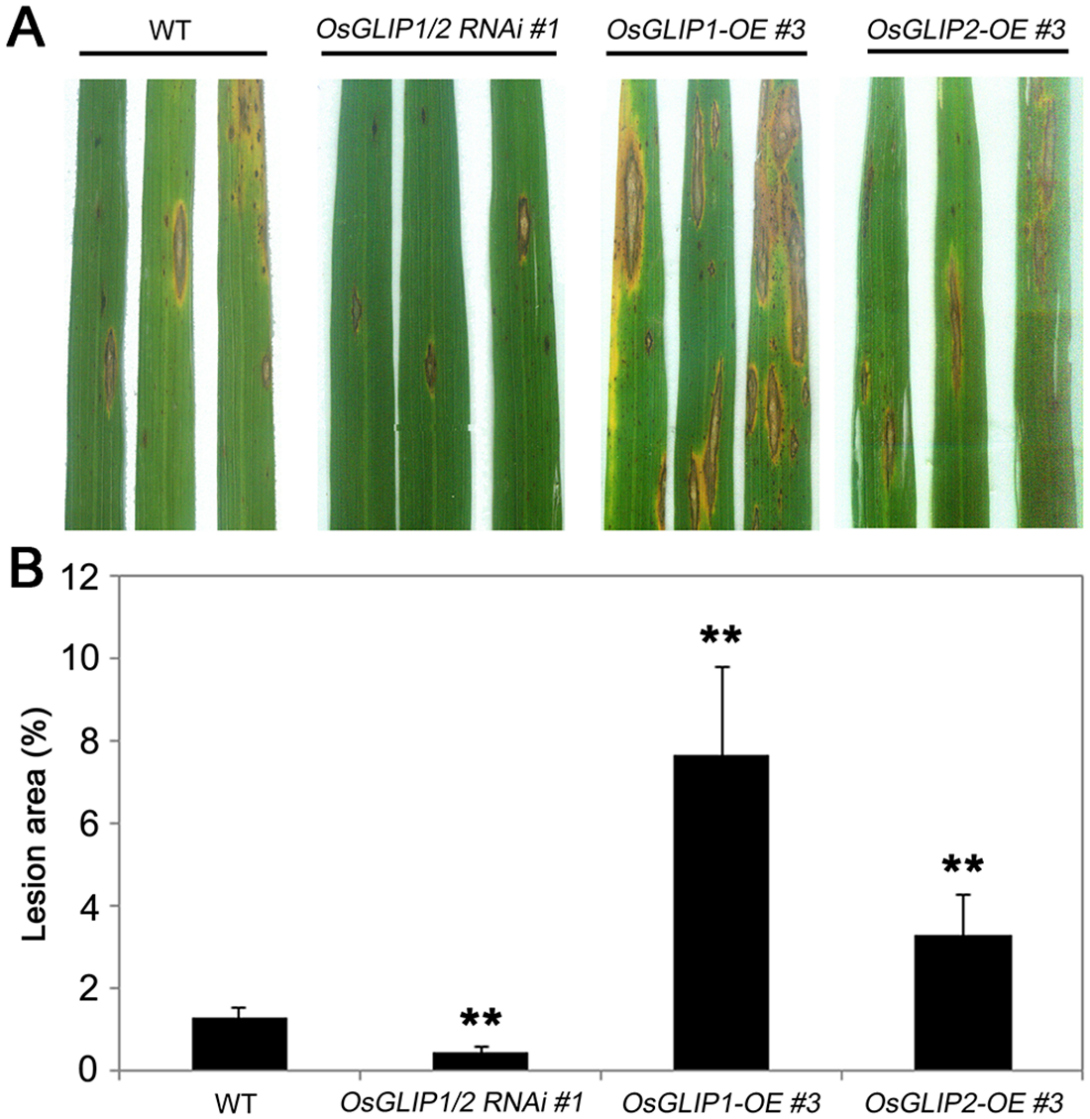
*OsGLIP* genes negatively affect resistance to rice blast. (A) Eight-week-old plants were inoculated with fungal pathogen *M*. *oryzae* by injection. Three leaves from the wild type and a representative line of *OsGLIP1-OE*, *OsGLIP2-OE* and *OsGLIP1/2*-*RNAi* transgenic plants were shown at 7 dpi. (B) Disease index of *M*. *oryzae* in the infected leaves of the wild-type and transgenic plants. Data are shown as relative lesion area compared to the whole leaf. Student’s *t*-test, ** *P* < 0.01.

We further investigated the roles of OsGLIP1 and OsGLIP2 in disease resistance against fungal blast (*M*. *oryzae*). *OsGLIP1* and *OsGLIP2* transgenic plants (three independent transgenic lines for each transgene) were inoculated with *M*. *oryzae*. Compared to the wild type, rice blast infection was strongly inhibited in *OsGLIP1/2*-*RNAi* plants, while the *OsGLIP1-OE* and *OsGLIP2-OE* plants exhibited more severe symptoms than the wild type (Fig 6A). We further compared the disease lesion sizes. Overexpression of *OsGLIP1* and *OsGLIP2* increased the lesion size to approximately 7 and 3 times, respectively, of the wild type. In contrast, the lesion area on OsGLIP1/2-*RNAi* leaves reduced to ~30% of wild-type plants (Fig 6B). Therefore, these pathogen inoculation assays again indicate that *OsGLIP1* and *OsGLIP2* negatively affect rice resistance to various pathogens.

### Differential expression patterns of *OsGLIP1* and *OsGLIP2* in plants

**Fig 7:**
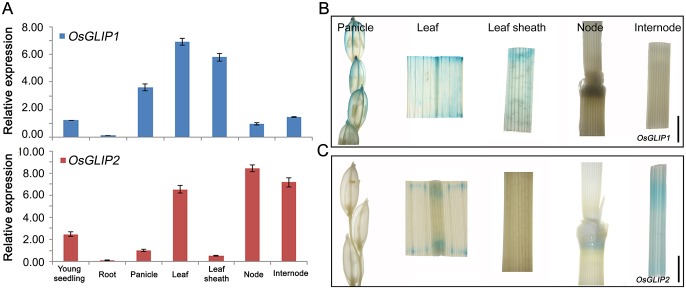
Expression patterns of *OsGLIP1* and *OsGLIP2*. (A) Tissue-specific expression of *OsGLIP1* and *OsGLIP2* in different tissues detected by qRT-PCR. The expression level of *OsGLIP1* was normalized to that of young seedling and *OsGLIP2* to panicle. Data shown are means ±SD from three biological replicates. (B, C) GUS staining of *pOsGLIP1*::*GUS* and *pOsGLIP2*::*GUS* transgenic plants revealed *OsGLIP1* and *OsGLIP2* expression in panicle, leaf, leaf sheath, node and internode of heading plants. Scale bars = 0.5 cm.

Given the involvement of OsGLIP1 and OsGLIP2 in lipid metabolism and rice disease resistance, we were then interested in their spatiotemporal expression patterns in plants. Quantitative RT-PCR analysis showed that both *OsGLIP1* and *OsGLIP2* transcripts are detected in various rice tissues. *OsGLIP1* expressed highly in leaves and sheaths, while *OsGLIP2* mainly expressed in nodes, internodes and leaves (Fig 7A). To further determine their expression patterns, the promoters of *OsGLIP1* and *OsGLIP2* were used to drive β-glucuronidase (GUS) expression. Consistent with the qRT-PCR assay, GUS staining of transgenic plants expressing *pOsGLIP1*::*GUS* and *pOsGLIP2*::*GUS* revealed that *OsGLIP1* is expressed in leaf, leaf sheath and young lemma (Fig 7B), while *OsGLIP2* is expressed in node and internode (Fig 7C), demonstrating that *OsGLIP1* and *OsGLIP2* exhibited differential but complementary expression patterns.

### Subcellular localization of OsGLIP1 and OsGLIP2

**Fig 8:**
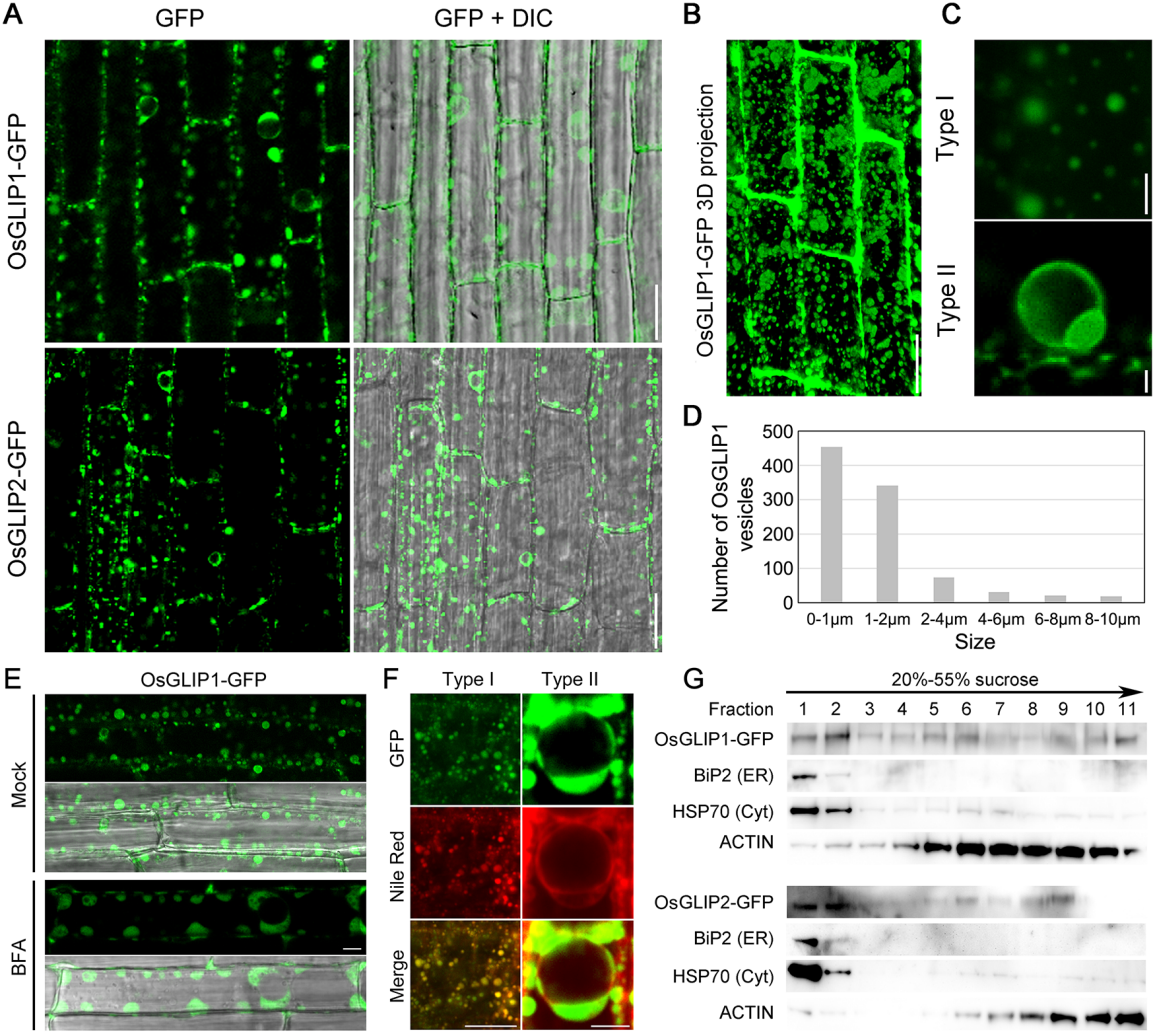
Subcellular localization of OsGLIP1 and OsGLIP2. (A) Localization of OsGLIP1-GFP and OsGLIP2-GFP in the root cells of transgenic plants. OsGLIP1 and OsGLIP2 were fused with GFP and expressed under the control of the maize *Ubiquitin* (*Ubi1*) promoter in the stable transgenic rice plants. Scale bars = 20 μm. (B) A 3D projection of OsGLIP1-GFP fluorescence signals. Scale bar = 20 μm. (C) Images of OsGLIP1-GFP labelled vesicle-like structures (Type I and Type II). Scale bar = 2 μm. (D) Size distribution of OsGLIP1-GFP labelled vesicles. The size of vesicles is represented by the diameter. (E) The localization of OsGLIP1-GFP in response to BFA treatment, in comparison with mock treatment. Scale bar = 20 μm. (F) Co-localization of OsGLIP1-GFP with Nile Red that labels lipid droplets. Scale bars = 20 μm (left) or 5 μm (right). (G) Distribution of OsGLIP1-GFP and OsGLIP2-GFP proteins in fractionated membranes of two-week-old rice seedlings. Microsomal membranes were fractionated on linear 20% to 55% (w/v) sucrose gradients. Equal volumes of protein samples were separated on SDS-PAGE gel and analysed by immunoblot using antibodies specific for GFP (GLIP1/2-GFP), BiP2 (ER), HSP70 (cytoplasm) and ACTIN. Note that OsGLIP1 and OsGLIP2 mainly localize to the lipid bodies (cytoplasm) and the ER.

Arabidopsis GLIP1 and Tanacetum TcGLIP have been shown to be secreted into the intercellular space when transiently expressed in onion epidermal [17,21]. However, the localization of most lipases in plant cells still remains elusive. To investigate the subcellular localization of OsGLIP1 and OsGLIP2, both proteins were fused with green fluorescent protein (GFP) and expressed under the control of the maize *Ubi1* promoter in stable transgenic rice. *Xoo* inoculation analysis indicated that *OsGLIP1-GFP* and *OsGLIP2-GFP* transgenic plants were more susceptible than the wild type, suggesting that the fusion proteins are functional equivalents to the wild-type proteins (S4 Fig). Localization of the fusion proteins were then visualized using confocal microscopy, and showed similar localization patterns. Both OsGLIP1-GFP and OsGLIP2-GFP proteins were observed principally in vesicle-like structures throughout the cytoplasm (Fig 8A). After plasmolysis, the OsGLIP-containing vesicles were mainly detected adjacent to the plasma membrane (PM) but some fluorescence signals remained in the apoplast (S5A and S5B Fig). To determine the nature of the apoplastic signals, high- or super- resolution microscopy would be required to test if OsGLIP1-GFP and OsGLIP2-GFP are normally secreted.

A 3D projection of OsGLIP1-GFP fluorescence signals indicated that the distribution of these OsGLIP-containing vesicles were not discrete but connected with each other by endomembrane systems (Fig 8B and S5C Fig). The size and morphology of these vesicles were found to be variable. Most of them are small and highly mobile (Type I) (Fig 8C, S1 Movie). There are also few much larger vesicles that are relatively static (Type II) (Fig 8C). Measurement of the vesicle size revealed that the diameter could be up to 10 μm but the majority of them were < 2 μm (Type I) (Fig 8D). The continuous trafficking of these small OsGLIP vesicles in the cytoplasm led us to examine their response to the treatment of brefeldin A (BFA), a specific vesicle trafficking inhibitor. We found BFA incubation resulted in aggregation of OsGLIP1-GFP fluorescence signals into big BFA compartments (Fig 8E). As BFA specifically blocks exocytosis but does not affect endocytosis, the sensitivity of OsGLIP vesicles to BFA treatment suggest that OsGLIP proteins might undergo active recycling.

The molecular nature of OsGLIP1 and OsGLIP2 as lipases and their vesicle localization pattern are reminiscent of lipid droplets. To directly verify this hypothesis, root cells expressing OsGLIP1-GFP were stained with Nile Red, a dye that specifically labels lipid bodies. The lipid staining assay revealed that in both small and large OsGLIP vesicles, GFP signals could overlap very well with the red signals from Nile Red (Fig 8F). The co-localization of OsGLIP1-GFP and OsGLIP2-GFP with Nile Red staining was further confirmed in rice protoplasts (S6 Fig). To further investigate the intracellular distribution of OsGLIP proteins, we carried out sucrose density gradient centrifugation assay. Microsomal membranes were prepared from OsGLIP1-GFP and OsGLIP2-GFP seedlings, fractionated on sucrose gradient (20% to 55%), and detected by Western blot analysis. We found that while both proteins are present in all membrane fractions, a majority of OsGLIP1-GFP and OsGLIP2-GFP proteins co-fractionate with the ER marker, BiP2 (Fig 8G). Taken together, these results suggest that OsGLIP proteins localize to endomembrane trafficking system as well as lipid droplets.

### Proper intracellular localization is a prerequisite for OsGLIP1 function in immune inhibition

**Fig 9:**
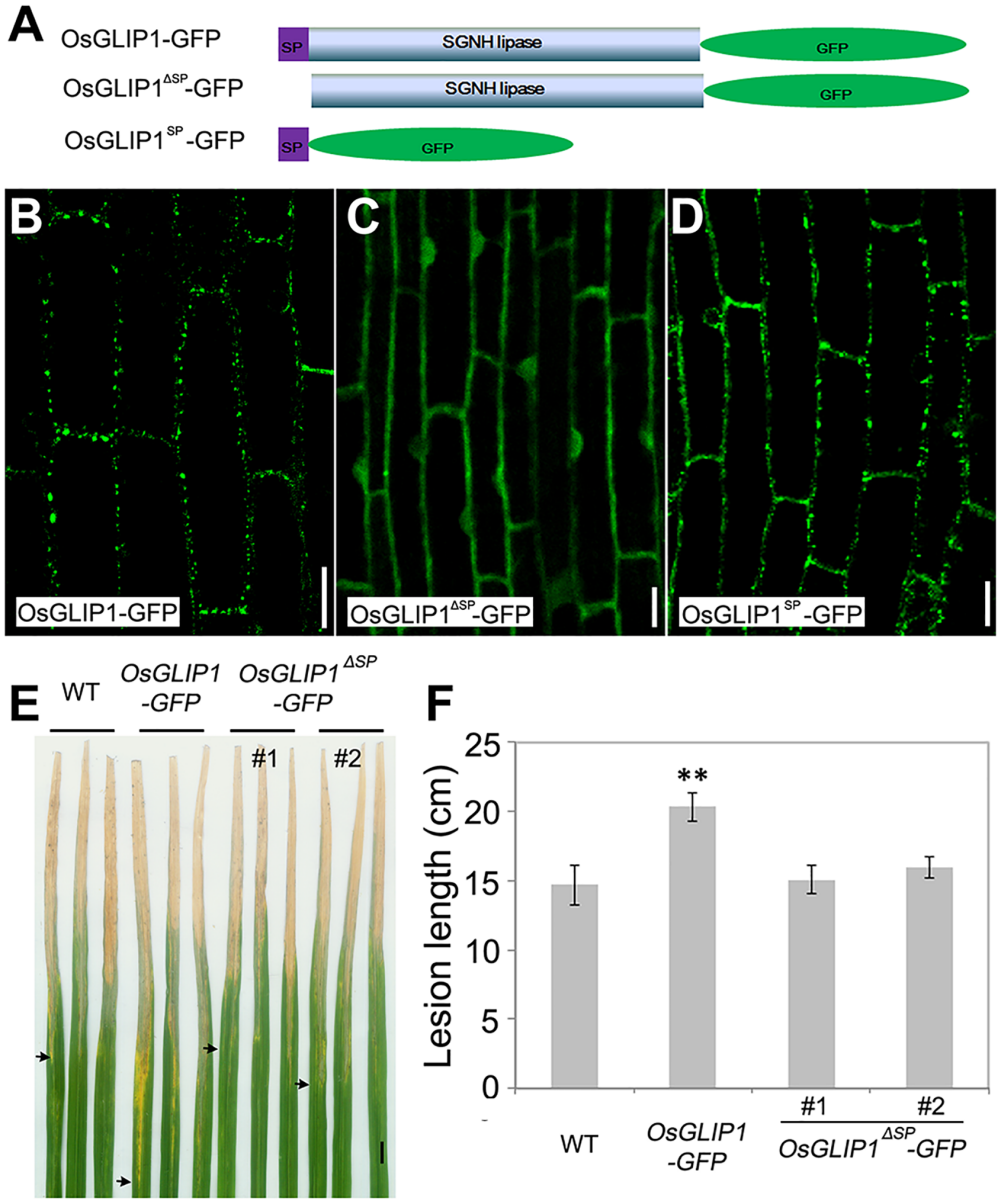
Proper intracellular localization is essential for OsGLIP1 function in rice defense responses. (A) Schematic diagram shows full-length OsGLIP1 with its signal peptide (SP) and the truncated protein without SP (OsGLIP1^ΔSP^-GFP) or SP alone (OsGLIP1^SP^-GFP) fused with GFP. (B-D) Subcellular localization of OsGLIP1 -GFP (B) OsGLIP1^ΔSP^-GFP (C) and OsGLIP1^SP^-GFP (D) proteins in root cells of transgenic plants. Note that removing of the signal peptide (SP) abolished OsGLIP1-GFP ER and lipid body targeting, while the SP alone was sufficient for the subcellular compartment targeting. Scale bars = 20 μm. (E, F) Deletion of the signal peptide attenuated OsGLIP1 action in suppressing plant immunity. Lesions (E) and lesion lengths (F) of representative *OsGLIP1-GFP* and *OsGLIP1*^*ΔSP*^*-GFP* transgenic plants inoculated with *Xoo*. Note that the OsGLIP1-GFP fusion protein also suppressed rice defense, while OsGLIP1^ΔSP^-GFP lost its immune inhibition capacity. Arrows indicate bottoms of lesions. Data are shown as means ± SD (*n* > 10). Scale bar in (E) = 1cm. Student’s *t*-test, **P < 0.01.

Proteins function with special subcellular localization, and it has been shown that the signal peptides of GDSL lipases are crucial for lipase localization [17,21]. Both OsGLIP1 and OsGLIP2 contain a typical signal peptide at the N-terminus with 29 and 35 aa, respectively. To evaluate the roles of the signal peptides in OsGLIP protein localization as well as biological functions, we generated two constructs, OsGLIP1^ΔSP^-GFP (without the signal peptide) and SP-GFP (the signal peptide alone), and transformed them into wild-type plants (Fig 9A). Confocal microscopy analysis revealed that the deletion of the OsGLIP1 signal peptide completely abolished its ER and lipid body localization, leading to OsGLIP1^ΔSP^-GFP localized ubiquitously in the cell including cytoplasm and nucleus (Fig 9B and 9C). By contrast, the signal peptide alone showed subcellular localization similar to the full length OsGLIP1 protein (Fig 9D), indicating that the signal peptide is necessary and sufficient for ER and lipid body targeting. Intriguingly, in contrast to the transgenic plants expressing full length OsGLIP1 protein compromising disease resistance, the transgenic plants expressing the truncated protein without signal peptide did not decrease disease resistance (Fig 9E and 9F), suggesting that the proper localization is critical for OsGLIP1 function in inhibiting plant immunity.

### MGDG and DGDG are involved in the inhibition of rice disease resistance

**Fig 10:**
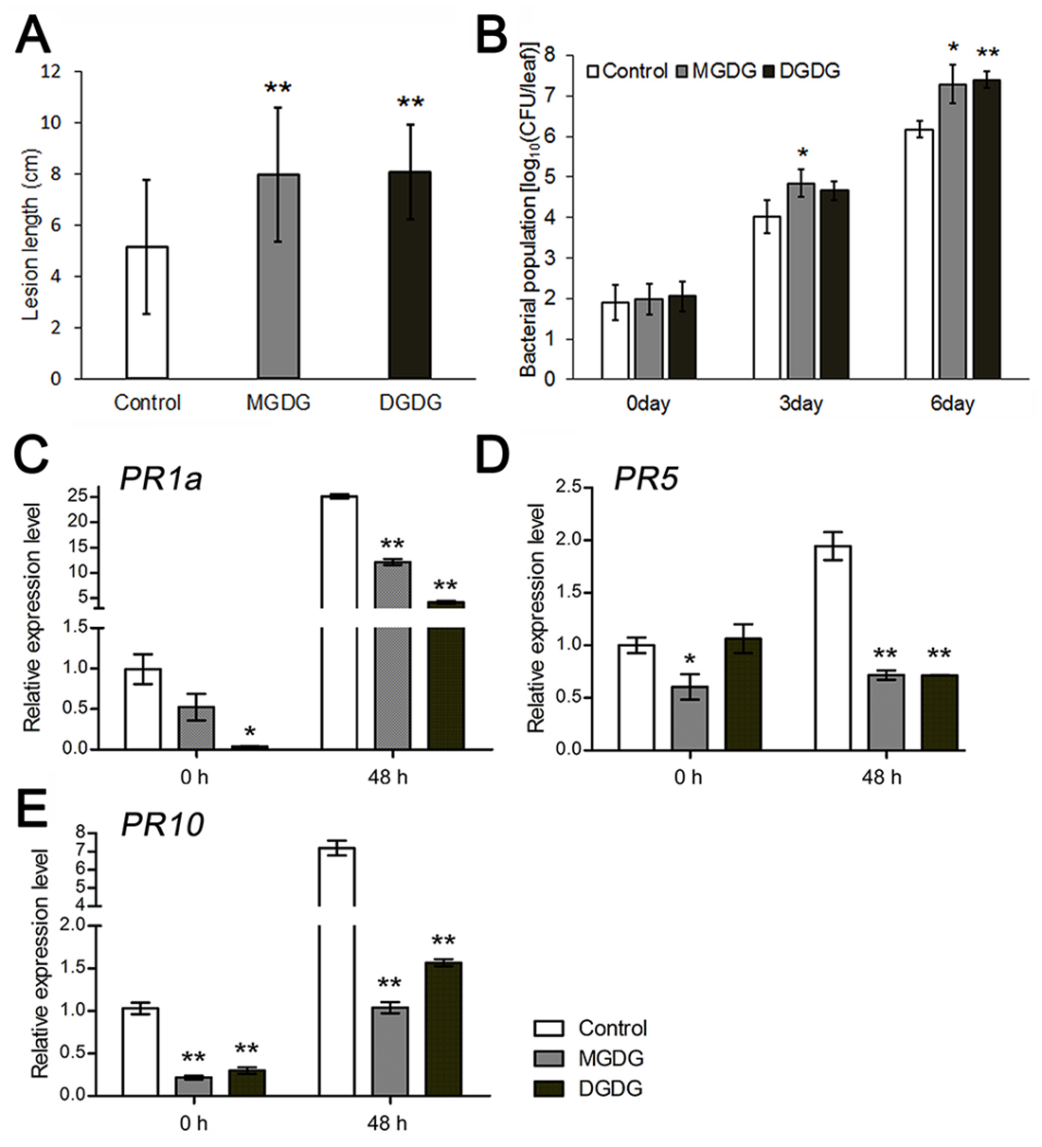
Exogenous feeding of MGDG and DGDG impairs rice disease resistance. (A) Two-week-old seedling leaves were cultured in liquid medium supplemented with MGDG or DGDG (100 μM with 0.1% Tween-20) for 24 hours. The treated plants were extensively washed and then inoculated with *Xoo* (strain PXO99A). Lesion lengths of MGDG and DGDG-fed plants were measured at 10 dpi with three biological replicates (> 10 plants each replicate). Data are shown as means ± SD from three biological replicates. (B) Bacterial growth in the MGDG and DGDG-fed plants at 0, 3 and 6 dpi, with mock treatment as control. Data are shown as means ± SD from three biological replicates. (C-E) Relative expression levels of *PR* genes *PR1a* (C), *PR5* (D) and *PR10* (E) in MGDG/DGDG treated leaves at 0 and 48 hpi. Data are shown as means ± SD from three biological replicates (*n* = 3). Student’s *t*-test, *P<0.05, **P < 0.01 (A to E).

MGDG and DGDG are abundant galactolipids present in thylakoid membranes and photosynthesis system I and II, which is crucial for photosynthetic efficiency and plant development [30,31]. Interestingly, recent reports also showed that both MGDG and DGDG are required for the induction of SAR non-redundantly in Arabidopsis. DGDG contributes to NO and SA biosynthesis involved in defense responses, while MGDG promotes the biosynthesis of AzA and G3P that function downstream of NO [11,32]. In our current work, we found that the levels of both MGDG and DGDG were increased in *OsGLIP1* and *OsGLIP2* overexpression plants (Fig 3D and 3F). To investigate the roles of MGDG and DGDG in rice defense, we performed feeding assays. Exogenous application of MGDG and DGDG increased the levels of both lipids in the leaves fed (S7 Fig), and importantly they also facilitated the growth of *Xoo* in rice leaves in dependent inoculation experiments (Fig 10A and 10B). In addition, the basal level of *PR* gene expression as well as pathogen-induced *PR* gene upregulation were both compromised in MGDG and DGDG treated plants (Fig 10C–10E), suggesting that MGDG and DGDG likely play a negative role in rice immunity.

## Discussion

Plant immune response is an energetically costly process that involves a rapid burst of ROS and programmed cell death [33]. To reduce the trade-off cost of defense over growth, a number of immune suppressors have been deployed such as rice NRR (an NPR1 interacting protein), WRKY transcription factors and *SPL11* and *EBR1* encoded E3 ubiquitin ligases, to compromise defense response thus maximizing fitness [34–38]. In this work, we have shown that two close GDSL lipases, OsGLIP1 and OsGLIP2, negatively affect rice immunity by modulation of lipid homeostasis, adding to the regulatory network of host-pathogen interaction and plant defense activation.

### OsGLIP1 and OsGLIP2 modulates lipid metabolism

GDSL lipases belong to a newly classified lipase family [25]. Although there are more than 100 GDSL lipases predicted in the rice genome [22,23], only two of them, WDL1 (WILTED DWARF AND LETHAL 1) and GER1 (GDSL CONTAINING ENZYME RICE 1), have been functionally studied, which regulate cuticle formation and coleoptile elongation, respectively [39,40]. In this study, we showed that both OsGLIP1 and OsGLIP2 exhibited lipase activities that could hydrolyze common lipid substrates (Fig 2), as reported for other GDSL lipases in diverse plant species [17,19]. Plant GDSL lipases have been implicated in both developmental and physiological processes; however, only TcGLIP from *Tanacetum cinerariifolium* has been shown to catalyze the ester-forming reaction for pyrethrin synthesis [21]. While the *in vivo* substrates and products of most GDSL lipases still remain enigmatic.

Lipid profiling has enabled us to compare the composition and abundance of individual lipid species in OsGLIP transgenic plants with the established methods [41,42]. We found that the overexpression of *OsGLIP1* and *OsGLIP2* increased the levels of MGDG and DGDG, two abundant galactolipids present in plant cells and crucial for photosynthetic efficiency and plant development [30,31]. MGDG and DGDG are produced by the galactosylation of diacylglycerol (DAG) [43]. In eukaryotic cells, DAG is mainly derived either from PA through the action of phospholipases [44], or from TAG by TAG lipases [45]. Although DAG levels only showed slight difference in *OsGLIP1* and *OsGLIP2* transgenic plants, the amount of its precursors TAG and PA exhibited significant changes. In *OsGLIP1-OE* plants, the amount of TAG was reduced; while in *OsGLIP2-OE* plants, the PA level was strongly decreased, suggesting that OsGLIP1 might act on TAG while OsGLIP2 is probably involved in PA hydrolysis. Consistent with this hypothesis, simultaneous down-regulation of both genes led to increasing of both TAG and PA levels (Fig 9). In plant cells, the production of DAG from PA mainly takes place in the ER, and hydrolysis of TAG into DAG occurs in lipid droplets [44,46]. DAG is then rapidly imported into plastid membranes for MGDG and DGDG synthesis [46,47]. Subcellular localization of both OsGLIP1 and OsGLIP2 into lipid bodies as well as the ER correlates with their potential roles in lipid metabolism. Further biochemical assays to dissect the catalytic functions of recombinant OsGLIP proteins on various lipid substrates would provide invaluable insights into the activity of the lipases.

### OsGLIP1 and OsGLIP2 are negative regulators of rice immunity

Recent studies are beginning to reveal roles of GDSL lipases in plant defense responses [17–21]. For instance, Arabidopsis GDSL lipases GLIP1 and GLIP2 exhibit antimicrobial activities and play positive roles in defense [17,19]. By contrast, virus-induced silencing of *CaGLIP1* in pepper resulted in enhanced resistance to bacterial pathogen *Xanthomonas campestris* pv. *vesicatoria* (*Xcv*), while overexpression of this gene in Arabidopsis increased plant susceptibility to both bacterial and fungal pathogens, indicating that *CaGLIP1* is an immune suppressor [20]. We proposed that OsGLIP1 and OsGLIP2 are suppressors of rice immunity based on both genetic and molecular evidence. Firstly, overexpression of *OsGLIP1* and *OsGLIP2* compromised plant resistance to both bacterial and fungal pathogens. Secondly, down-regulation of both genes significantly increased disease resistance. Finally, the up-regulation of *PR* genes in response to pathogen infection was suppressed in overexpression plants but substantially enhanced in *OsGLIP1/2*-*RNAi* plants compared to wild-type plants. With this scenario, *OsGLIP*1 and *OsGLIP2* were down-regulated in response to pathogen infection and SA/BTH treatment. The dual roles of GDSL lipases as either positive or negative regulators of plant defense in different species reflect their broadly diverse catalytic activities in lipid metabolism, and substrates and/ or products of GDSL lipases likely function differentially in diverse plants [16], given that structural and biochemical analysis of bacteria GDSL lipases has revealed a flexible active site compatible with different substrates [48].

The altered disease resistance in *OsGLIP* transgenic plants was associated with significant changes of TAG, PA, MGDG and DGDG. Although the role of TAG in plant immunity remains to be elucidated, increasing evidence has implicated PA as an important regulator of plant immune responses. It has been shown that PA is rapidly produced upon pathogen infection/elicitor treatments. The elevated levels of PA subsequently induced ROS production, SA accumulation and defense activation [49–51]. Given the positive role of PA in plant immune responses, the increased/ decreased level of PA in *OsGLIP1/2*-*RNAi*/*OsGLIP-OE* plants might contribute to enhanced/compromised basal resistance to pathogens. More interestingly, exogenous application of MGDG and DGDG compromised rice disease resistance (Fig 10). Similarly, resistance was also compromised in *OsGLIP1-OE* and *OsGLIP2-OE* plants in which the abundance of MGDG and DGDG was increased. Consistent with these observations, both MGDG and DGDG contents have been shown to decrease in rice leaves infected by *M*. *oryzae* [52]. These findings together suggest that MGDG and DGDG most likely function as suppressor of rice immune responses. This is in contrast to the positive roles of MGDG and DGDG in Arabidopsis disease resistance via the establishment of SAR [11,32]. However, the SAR signaling pathway is not well documented in monocot plants such as rice [53]. Compared to the basal levels of SA in Arabidopsis, rice contains much higher levels of endogenous SA even in absence of pathogen infection [53,54]. Therefore, MGDG and DGDG, as well as GDSL lipases, would presumably play divergent roles in plant defense across different species.

### Sub-functionalization of OsGLIP1 and OsGLIP2

Rice GDSL lipases constitute a large gene family which can be divided into four clades and twelve subclades, with each subclade consisting of sister gene pairs. Phylogenic analysis suggested that gene duplication plays a major role in the expansion of these GDSL genes [22]. Duplicated genes usually undergo sub-functionalization in protein activities, cellular localizations and/ or gene expression patterns [55]. For example, duplicated *Populus* Class III peroxidases exhibit divergent subcellular localization in either the cell wall or vacuole [56]. Rice cell-wall invertase genes *GIF1* and *OsCIN1* are a pair of duplicate genes required for seed development as well as disease resistance, *GIF1* is mainly expressed in the ovular vascular tissue while *OsCIN1* transcript is detected in pericarp and endosperm [57]. Similarly, we found that *OsGLIP1* and *OsGLIP2* also exhibited divergent expression patterns. The differential but also complementary expression patterns of *OsGLIP1* and *OsGLIP2* likely help to establish an elaborate metabolic cascade to regulate plant immunity throughout different tissues and development stages. Biochemical and transgenic analysis indicated that OsGLIP1 and OsGLIP2 proteins display similar catalytic activities and biological functions. However, comparison of lipid profiles revealed that the two lipases might have subtle difference in substrate preference *in planta*. Therefore, OsGLIP1 and OsGLIP2 might constitute a pair of genes with divergent expression patterns and different catalytic properties, and function synergistically to regulate rice immunity via modulation of lipid homeostasis in a temporal and spatial context.

## Materials and methods

### Plant materials, growth conditions and chemical induction

All the experiments were performed with rice variety Taipei 309 (TP309). Plants were grown in the paddy field under natural growing conditions. For experiments with seedlings, such as chemical treatments, two-week-old plants were grown in a growth chamber under conditions of 12-h day, 28°C, 80% RH followed by 12-h night, 26°C, 60% RH. BTH and SA treatments were performed according to the previous study [58]. BTH (in 0.5% acetone and 0.05% Tween 20) and SA (in 0.01% Tween 20) were sprayed onto leaves (1 ml/plant). Mock treatments were done by spraying the solvents only.

### Gene cloning and plasmid construction

*OsGLIP1* (1197 bp) and *OsGLIP2* (1212 bp) cDNA were isolated according to rice genome information. The cDNAs were amplified further using primers GLIP1-OE-F/R and GLIP2-OE-F/R and inserted into the binary vector pUN1301 to form the *OsGLIP1-OE* and *OsGLIP2-OE* overexpression constructs. For RNAi cloning, we chose the conserved sequence of *OsGLIP1* and *OsGLIP2* coding region and amplified a short fragment with primers GLIP1/2-CK303-F (with *Bam*HI site) and GLIP1/2-CK303-R (*Kpn*I and *Spe*I sites). The fragments were introduced into vector pTCK303 [59]. The transgenic constructs were transformed into TP309 by the Agrobacterium-mediated transformation method. More than 30 independent transgenic lines were obtained for each construct. The primers for cloning used in this study are detailed in S1 Table.

### Phylogenetic analysis

Protein sequences of GDSL lipase family members in rice and other organisms were retrieved from NCBI (http://www.ncbi.nlm.nih.gov). Multiple sequence alignments of protein were done in Clustal X (1.83). A phylogenetic tree of aligned sequence was constructed by MEGA (version 4.0.2) using the neighbor-joining method with the following parameters: Poisson correction, complete deletion, and bootstrap (1000 replicates, random seed).

### Pathogen inoculation and disease assay

For *Xoo* resistance assay, eight-week-old plants were inoculated with Philippine race P6 (PXO99A) by the leaf-clipping method as previously described [60,61]. Lesion length was recorded 14 days after inoculation. More than thirty leaves were used for statistical analysis. For *Xoo* growth curve, 20 cm of leaf tissue from the top was ground and resuspended in 10 ml sterile water to collect bacteria. The suspensions were diluted accordingly and plated on peptone sucrose agar (PSA) plates containing 15 mg/l cephalexin. Bacteria clones were counted after 3 days incubation at 28°C.

The injection-inoculation method was employed for *M*. *oryzae* infection assay as described in [62]. Each tiller was injected with 0.1 ml mixed blast spore suspensions. Lesions developed on leaves were recorded 7 days after inoculation. Disease index was calculated by measuring the percentage of the lesion area (Lesion area/ Leaf area).

### Protein purification and lipase activity assay

The OsGLIP1 and OsGLIP2 coding regions without N-terminal signal peptide sequences were amplified by primer GLIP1Δ29-enzyme-F/R and GLIP2Δ35-enzyme-F/R, respectively (S1 Table). The fragments were subsequently cloned into the pGEX-4T-3 vector (GE Healthcare). Constructs were transformed into *Escherichia coli* BL21 (DE3) cells and the expression of the recombinant proteins was induced by the addition of 0.2 mM isopropylthio-β-galactoside. Cells were harvested 6 hours after induction, suspended in PBS buffer and sonicated. The supernatants (lysates) were then purified using GST-tag beads (GE Healthcare). The purified proteins were confirmed by SDS-PAGE gel and stored at –70°C.

Lipase activity was measured as described [63]. The enzyme reaction mixture contained 0.5 M HEPES, pH 6.5, 1 mM substrate (p-nitrophenyl acetate or p-nitrophenyl butyrate), and aliquots of recombinant proteins (2 to 4 μg) and was incubated for 60 min at 30°C. Absorbance was measured at 405 nm every 5 min for 60 min.

Western blot of the fusion proteins (OsGLIP1-GFP and OsGLIP2-GFP) was performed with an anti-GFP antibody, signals were visualized by using ECL systems, and images were captured using the Tanon-5200 Chemiluminescent imaging system (Tanon).

### Tissue-specific expression of *OsGLIP1* and *OsGLIP2*

A 3.3-kb or 2.9-kb promoter region of *OsGLIP1* or OsGLIP2 was amplified using primers GLIP1-promoter-F/R and GLIP2-promoter-F/R (S1 Table), respectively, and then inserted into the expression vector 1300-GUS-Nos to generate pOsGLIP1::GUS and pOsGLIP2::GUS. The constructs were transformed into TP309 to generate fusion reporter gene transgenic plants. For GUS staining, various tissues of pOsGLIP1::GUS and pOsGLIP2::GUS transgenic plants were incubated in a solution containing 50 mM NaPO_4_ buffer (pH 7.0), 5 mM K_3_Fe(CN)_6_, 5 mM K_4_Fe(CN)_6_, 0.1% Triton X-100, and 1 mM X-Gluc at 37°C.

### Subcellular localization of OsGLIP1 and OsGLIP2

The full-length coding regions of OsGLIP1 and OsGLIP2 were amplified using primers GLIP1-GFP-F/R and GLIP2-GFP-F/R (S1 Table) and inserted into pUN1301 with in-frame fusion with GFP. The resulting constructs were transformed into TP309 to produce GFP fusion plants. GFP signals in roots were visualized using confocal laser scanning microscope (Zeiss LSM510 and Leica TCS SP8). For observation of protein localization in protoplasts, rice protoplasts were isolated from leaf sheaths of the OsGLIP1-GFP and OsGLIP2-GFP transgenic plants according to a previously reported method [64].

### Quantitative real time-PCR

Total RNA was extracted from different tissues using TRIzol reagent according to the manufacturer’s instructions (Invitrogen). For RT–PCR analysis, 2 μg RNA was reverse-transcribed into cDNA using oligo (dT) primer and SuperScript III reverse transcriptase (Invitrogen) and then used as templates for PCR with gene-specific primers. Quantitative RT–PCR analysis was performed using SYBR Premix Ex Taq (TaKaRa) and gene-specific primers (S2 Table).

### Lipid measurements

Lipid extraction was carried using modified Bligh & Dye’s protocol as previously described [65]. Leaves from six individual plants (eight-week-old) were mixed as one sample and five samples (5 replicates) from three independent transgenic lines of each transgene were used to normalize samples. All analyses were conducted using an Agilent 1260 HPLC system coupled with a triple quadrupole/ion trap mass spectrometer (5500Qtrap; SCIEX) in the electrospray ionization (ESI) mode under the following conditions: curtain gas = 20, ion spray voltage = 5500 V, temperature = 400°C, ion source gas 1 = 35, and ion source gas 2 = 35.

For normal phase (NP) LC/MS, polar lipid analysis was conducted as previously described [65]. Briefly, individual classes of polar lipids were separated by NP-HPLC with the use of a Phenomenex Luna 3μm-silica column (internal diameter 150 × 2.0 mm) under the following conditions: mobile phase A (chloroform: methanol: ammonium hydroxide, 89.5: 10: 0.5) and mobile phase B (chloroform: methanol: ammonium hydroxide: water, 55: 39: 0.5: 5.5). The gradient began with 95% mobile phase A for 5 min, followed by linear reduction to 60% mobile phase A over 7 min. The gradient was held for 4 min, and mobile phase A was then further reduced to 30% and held for 15 min. The column was then reconditioned with the initial gradient for 5 min.

MRM transitions were constructed for comparative analysis of various polar lipids. Quantification of individual lipid species were carried out by referencing to spiked internal standards; namely PC-14:0/14:0, PC34:1-d31, PE-14:0/14:0, PE34:1-d31, PS-14:0/14:0, PA-17:0/17:0, PG-14:0/14:0, which were obtained from Avanti Polar Lipids (Alabaster, AL) and LIPID MAPS. Dioctanoyl phosphatidylinositol (PI) (16:0-PI) was purchased from Echelon Biosciences, Inc. (Salt Lake City, UT) and used together with PI34:1-d31 (LIPID MAPS) for PI quantitation. Qualitative deuterated lipid standards from LIPID MAPS were pre-corrected based on available quantitative lipid standards prior to their use for quantitation.

For reverse phase LC/MS, glycerol lipids [DAGs and TAGs] were analyzed using a modified version of reverse phase (RP)-HPLC/ESI/MS/MS as reported previously [65]. In brief, separation of the aforementioned lipids was carried out on a Phenomenex Kinetex 2.6μm-C18 column (internal diameter 4.6×100 mm) using an isocratic mobile phase chloroform:methanol:0.1M ammonium acetate (100:100:4) at a flow rate of 160 μl/min for 20 min. Based on neutral loss MS/MS techniques, the levels of TAGs were calculated relative to the intensity of spiked d5-TAG 48:0 internal standard (CDN Isotopes), while DAG species were quantified using 4ME 16:0 diether DG as an internal standard (Avanti Polar Lipids). Separation of galatolipids (MGDGs and DGDGs) were conducted using Phenomenex Kinetex 2.6μm-C18 column (internal diameter 4.6 × 100 mm) with an isocratic gradient of chloroform:methanol: 2% of 50 mmol/L sodium acetate (49: 49: 2) at a flow rate of 160 μl/min for 25 min as reported previously [42]. MRM (multiple reaction monitoring) and SIM (selective ion monitoring) transitions for lipids presented in this study were provided in S3 Table.

### MGDG and DGDG feeding assays

MGDG and DGDG (Avanti Polar Lipids, Inc) were dissolved in acetone respectively, and then diluted with water to 100 μM with 0.1% Tween-20. Leaves of two-week-old seedlings (TP309) were soaked in the MGDG or DGDG solution for 24 hours, with mock (acetone diluted with a comparable amount of water with 0.1% Tween-20) solution as control. Leaves were immediately were washed extensively to remove residual solution. Treated seedlings were inoculated with *Xoo* (strain PXO99A) by the leaf-clipping method [60]. *Xoo* growth curve was assayed as described above.

To measure the content of MGDG and DGDG in fed leaves, leaves after feeding were extensively washed to remove residual solution. Lipid samples were prepared according to the methods described in [52]. In detail, fresh seedling leaves (0.15g) were powdered with liquid N_2_. A solution (1.5mL) of methanol: chloroform: water (1: 1: 0.74, v/v) containing 200 μM L^-1^ of butylated hydroxytoluene was then added to extract total lipids. Chloroform layer was then concentrated with an Eppendorf concentrator. Extracts were kept at -20°C in tubes filled with gaseous N_2_ to avoid oxidation and hydrogenation of the galactolipids. Extracts were dissolved in 200 μL acetonitrile: water: isopropanol (3: 2: 5, v/v, 5mM ammonium formate), then diluted by 20 times for measuring.

Liquid chromatography–high resolution mass spectrometry (LC–HRMS) was applied to determine the contents of MGDG and DGDG. HRMS data was obtained from a Q-Exactive Quadrupole Orbitrap mass spectrometer (Thermo Fisher Scientific, Bremen, Germany) coupled to an Acquity Ultra Performance LC system (Waters). An Acquity UPLC BEH C18 column (2.1×50mm, 1.7 μm) was used at 40°C. The lipid extract of 1 μl was injected at 12°C. Acetonitrile:water (60/40, v/v, 10mM ammonium formate) and isopropanol:acetonitrile (90/10, v/v) were used as mobile phases A and B, respectively. The flow rate was 0.3 mL/min. The elution was performed with a 14 min gradient with 30% B at the beginning, then linearly increased from 30% B to 95% B in 8 min. After washing the column for 2 min with 95% B, the buffer was decreased to 30% B immediately and the column was reconditioned with initial gradient for 4 min. The MS was operated with HESI ion source in positive mode. The following conditions were used for HESI source: capillary voltage, 4.0 KV; capillary temperature, 320°C; sheath gas, 35.00 units; auxiliary gas, 5.00 units; probe heater temperature, 300°C; S-Lens RF level, 60.00. The mass spectrometer was run in full MS-ddMS2 mode. The full MS scan used the following settings: resolution, 70,000; AGC target, 3e6; scan range, 500–1200 m/z. The ms2 scan parameters: Loop count, 5; resolution, 17,500; AGC target, 1e5; max IT, 50 ms; isolation window, 1.0 m/z; normalized HCD collision energy, 15 eV and 35 eV. Standard DGDG and MGDG (Avanti Polar Lipids) were used to make standard curves. The resulting linear equation in our experiments for MGDG was Y = -3.34662 × 10^7^ + 4.12273 × 10^6^X, R^2^ = 0.9967; and for DGDG, Y = -1.39113 × 10^7^ + 5.6299×10^6^X, R = 0.9985, with X as amounts of MGDG or DGDG (μg), and Y as the integrated area.

### Sucrose density-gradient centrifugation

Two-week-old plants were used for membrane fractionation. Membrane fractionation was carried out by a protocol described previously [66,67] with some modifications. In brief, tissues were homogenized in cold buffer [30mM Tris, pH7.5, 150mM NaCl, 10mM EDTA, 20% glycerol with proteins inhibitor cocktail (Roche)], and were filtered through two layers of Miracloth (Merck) and then centrifuged at 10,000g for 10 min at 4°C. Supernatants were centrifuged again at 100,000g for 50 min at 4°C to pellet the membrane fraction. The resulting microsome pellets were resuspended in 0.5 mL of resuspension buffer [10mM Tris, pH7.5, 10mM EDTA, 10% sucrose with 1mM DTT and proteins inhibitor cocktail (Roche)]. Serial 20%, 25%, 30%, 35%, 40%, 45%, 50%, 55% (w/v) sucrose solutions were prepared with 20 mM HEPES (pH7.5), 1 mM EDTA, and were then added to ultracentrifuge tubes (Beckman SW14) one by one to form continuous gradients. Lipid samples (0.5 ml) were added and centrifuged at 100,000g for 12 h with an ultracentrifuge (Optima L-90K, Beckman). Fractions (1 ml) were collected from top to bottom and transferred to new tubes for protein assay.

### Accession numbers

Sequence data from this article can be found in the GenBank/EMBL database under the following accession numbers: KU350624 (OsGLIP1), NM_001063391 (OsGLIP2).

## Supporting information

S1 Table PCR primers used for cloning. (DOCX)

S2 Table Primers used for quantitative PCR in gene expression analysis. (DOCX)

S3 Table MRM (multiple reaction monitoring) and SIM (selective ion monitoring) transitions for lipid species analyzed. (DOCX)

S1 Fig Phylogenetic analysis of rice GDSL lipases. (A) Gene structure of OsGLIP1 and OsGLIP2. (B) A phylogenetic tree of GDSL lipases in rice. Protein sequences were aligned with CLUSTAL W, and the phylogenetic tree was constructed with MEGA4.
(PDF)

S2 Fig Relative expression levels of *OsGLIP1* and *OsGLIP2* in overexpression and RNAi plants. The expression of *OsGLIP1* and *OsGLIP2* in 9 independent *OsGLIP1-OE* (A) and *OsGLIP2-OE* (B) lines and 8 independent *OsGLIP1/2*-*RNAi* lines (C) was compared to the wild-type control with normalization to rice *Actin1* gene. Error bars, ± SD (n = 3).
(PDF)

S3 Fig Profiling of individual molecular species of lipids. The lipids, PC (A), PI (B), PE (C) and PG (D), are presented as the number of carbon atoms: total double bonds in the fatty acyl groups. Data are shown as means ± SD (n = 5) of mixed leaf samples from three representative transgenic lines. *P < 0.05 or **P < 0.01, by Student’s *t*-test and Bonferroni correction for multiple (three comparisons) tests.
(PDF)

S4 Fig Enhanced disease susceptibility in OsGLIP1-GFP and OsGLIP2-GFP transgenic plants. (A) Lesion lengths in the leaves of wild-type and independen*t OsGLIP1-GFP* and *OsGLIP2-GFP* transgenic plants. Plants at booting stage were infected with *Xoo*. Lesion length was calculated at 14 dpi. Data are shown as means ± SD (n > 10). Student’s *t*-test, **P < 0.01. (B) Western blot analysis with GFP antibodies confirmed the accumulation of the fusion proteins of OsGLIP1-GFP and OsGLIP2-GFP in the transgenic plants. Rubisco staining was used as loading control. (C) Bacterial growth during 8 days of inoculation in the representative transgenic lines. Data are shown as means ± SD (*n* = 3). Asterisks indicate significant difference in comparison with the wild-type control (Student’s *t*-test, *P < 0.05; ** *P* < 0.01).
(PDF)

S5 Fig Plasmolysis analysis of *OsGLIP1-GFP* and *OsGLIP2-GFP* transgenic plants cells. The root tips of transgenic plants were incubated in 25% sucrose to induce cell plasmolysis and then observed under confocal microscopy. (A, B) Confocal images of *OsGLIP1–GFP* (A) and *OsGLIP2–GFP* (B) after cell plasmolysis. Scale bars = 20 μm. (C) *OsGLIP1-GFP* root cells without plasmolysis to show the endomembrane systems that link OsGLIP1-GFP labelled vesicles. Scale bar = 20 μm.
(PDF)

S6 Fig Co-localization of OsGLIP1 and OsGLIP2 with Nile Red in rice protoplasts. Protoplasts were generated from seedlings of transgenic plants expressing OsGLIP1-GFP and OsGLIP2-GFP fusion protein. Note the overlapping of GFP green signals and Nile Red stained red signals, while OsGLIP1-GFP and OsGLIP2-GFP were not associated with chloroplasts. Scale bars = 10 μm.
(PDF)

S7 Fig The contents of MGDG and DGDG in fed leaves. Two-week-old rice leaves were fed with 100μM MGDG (A) and DGDG (B) for 24 hours and subsequently used for lipid extraction and measurements. Data are shown as means ± SD of four biological replicates. Student’s *t*-test. *P < 0.05.
(PDF)

S1 Movie Active movement of OsGLIP1-GFP labelled vesicles in rice root cells. (AVI)
