# Supplementary material for: GDSL lipases modulate immunity through lipid homeostasis in rice
Source: PLoS Pathog. 2017 Nov 13;13(11):e1006724. doi: 10.1371/journal.ppat.1006724 (PMC5703576; doi:10.1371/journal.ppat.1006724)
Supplement: S1 Table — (DOCX) [file ppat.1006724.s001.docx]

**S1 Table.** **PCR primers used for cloning**

| Primer | Sequence (5'-3') | Site |
| --- | --- | --- |
| GLIP1/2-CK303-F | GGATCCTTCGCGCTCTACCTGA | BamHI |
| GLIP1/2-CK303-R | GGTACCACTAGTGCAGAATGGGCG | KpnI SpeI |
| GLIP1-OE-F | GGATCCAGGAAAACGAAAGCCAT | BamHI |
| GLIP1-OE-R | GGTACCGGAGGGAAGGGAGTAT | KpnI |
| GLIP1-GFP-F | GGATCCAGGAAAACGAAAGCCAT | BamHI |
| GLIP1-GFP-R | GGTACCGAGCAGAATGGGCGGGTGGCAG | KpnI |
| SP(GLIP1)-GFP-F | GGATCCAGGAAAACGAAAGCCAT | BamHI |
| SP(GLIP1)-GFP-R | GGTACCGCCGGCGACGACGCCGACGA | KpnI |
| GLIP1Δ29-GFP-F | GGATCCATGGAGCATGGCGGCGGC | BamHI |
| GLIP1Δ29-GFP-R | GGTACCGAGCAGAATGGGCGGGTGGCAG | KpnI |
| GLIP1-promoter-F | CTGCAGCCTATGACTGGCTACTTCAGT | PstI |
| GLIP1-promoter-R | GTCGACTTTCGTTTTCCTGCACTCT | SalI |
| GLIP1Δ29-enzyme-F | GGATCCGAGCATGGCGGCGGCGGC | BamHI |
| GLIPΔ29-enzyme-R | CTCGAGAGCAGAATGGGCGGGTGGCA | XhoI |
| GLIP2-OE-F | GGTACCGAGCTGTGGCGTTGCG | KpnI |
| GLIP2-OE-F | GAGCTCGTTCTGCGGCTACACT | SacI |
| GLIP2-GFP-F | GGATCCGTGGCGTTGCGAGTG | BamHI |
| GLIP2-GFP-R | GGTACCGTGGTGCAGGATGGG | KpnI |
| GLIP2-promoter-F | AAGCTTATCAACTTGGTTTGC | HindⅢ |
| GLIP2-promoter-R | GTCGACGCGAGACAGTGGCAT | SalI |
| GLIP2Δ35-enzyme-F | GGATCCGGTGGCGTCGGCGGCGGC | BamHI |
| GLIP2Δ35-enzyme-R | CTCGAGTGGTGCAGGATGGGAGGGT | XhoI |
