# Supplementary material for: GDSL lipases modulate immunity through lipid homeostasis in rice
Source: PLoS Pathog. 2017 Nov 13;13(11):e1006724. doi: 10.1371/journal.ppat.1006724 (PMC5703576; doi:10.1371/journal.ppat.1006724)
Supplement: S2 Table — (DOCX) [file ppat.1006724.s002.docx]

**S2 Table.** **Primers used for quantitative PCR in gene expression analysis**

| Primer | Sequence (5'-3') | GenBank accession |
| --- | --- | --- |
| GLIP1-realtime-F | CCAAATTGCAAGTGGCGTCGAGAA |  |
| GLIP1-realtime-R | AGTCGGACTTGTTGCTGCTCTTGA |  |
| GLIP2-realtime-F | ACTTCGACTTCCTCAAGTCCATCG | AK107226 |
| GLIP2-realtime-R | AACAGCATGGCGTTGTAGTCGTTG |  |
| OsPR1a-F | CGTCTTCATCACCTGCAACTACTC | AJ278436 |
| OsPR1a-R | CATGCATAAACACGTAGCATAGC |  |
| OsPR1b-F | GGCAACTTCGTCGGACAGA | U89895 |
| OsPR1b-R | CCGTGGACCTGTTTACATTTTCA |  |
| OsPR5-F | CAACAGCAACTACCAAGTCGTCTT | X68197 |
| OsPR5-R | CAAGGTGTCGTTTTATTCATCAAC |  |
| OsPR10-F | CCCTGCCGAATACGCCTAA | D38170 |
| OsPR10-R | CTCAAACGCCACGAGAATTTG |  |
| Actin-F | TGTATGCCAGTGGTCGTACCA | X15865 |
| Actin-R | CCAGCAAGGTCGAGACGAA |  |
