# Supplementary material for: GDSL lipases modulate immunity through lipid homeostasis in rice
Source: PLoS Pathog. 2017 Nov 13;13(11):e1006724. doi: 10.1371/journal.ppat.1006724 (PMC5703576; doi:10.1371/journal.ppat.1006724)
Supplement: S3 Table — (DOCX) [file ppat.1006724.s003.docx]

| **S3 Table. MRM and SIM transitions for lipid species analyzed*** | | | | | | | |  | |  |
| --- | --- | --- | --- | --- | --- | --- | --- | --- | --- | --- |
| Lipid Species | Q1 | Q3 | Lipid Species | Q1 | Q3 | Lipid Species | Q1 | | Q3 | |
| MGDG 34:3 | 775.5 | 243 | DAG 16:1/16:1 | 582.5 | 311.2 | TAG 50:2 | 848.8 | | |  |
| MGDG 34:2 | 777.5 | 243 | DAG 16:2/16:0 | 582.5 | 313.2 | TAG 50:1 | 850.8 | | |  |
| MGDG 35:3 | 789.5 | 243 | DAG 16:1/16:0 | 584.5 | 311.2 | TAG 52:6 | 868.8 | | |  |
| MGDG 36:6 | 797.5 | 243 | DAG 18:2/16:1 | 608.5 | 313.2 | TAG 52:5 | 870.8 | | |  |
| MGDG 36:3 | 803.5 | 243 | DAG 16:2/18:0 | 610.5 | 341.2 | TAG 52:4 | 872.8 | | |  |
| DGDG 34:3 | 937.5 | 405 | DAG 16:0/18:2 | 610.5 | 337.2 | TAG 52:3 | 874.8 | | |  |
| DGDG 34:2 | 939.5 | 405 | DAG 16:1/18:1 | 610.5 | 339.2 | TAG 52:2 | 876.8 | | |  |
| DGDG 35:3 | 951.5 | 405 | DAG 16:1/18:0 | 612.5 | 341.2 | TAG 52:0 | 880.8 | | |  |
| DGDG 36:6 | 959.5 | 405 | DAG 16:0/18:1 | 612.5 | 339.2 | TAG 54:9 | 890.8 | | |  |
| DGDG 36:3 | 965.5 | 405 | DAG 16:0/18:0 | 614.5 | 341.2 | TAG 54:8 | 892.8 | | |  |
| PA 32:2 | 643.5 | 153 | DAG 18:3/18:3 | 630.5 | 335.2 | TAG 54:7 | 894.8 | | |  |
| PA 32:1 | 645.5 | 153 | DAG 18:2/18:2 | 634.5 | 337.2 | TAG 54:6 | 896.8 | | |  |
| PA 34:3 | 669.5 | 153 | DAG 18:2/18:1 | 636.5 | 339.2 | TAG 54:5 | 898.8 | | |  |
| PA 34:2 | 671.5 | 153 | DAG 18:1/18:1 | 638.5 | 339.2 | TAG 54:4 | 900.8 | | |  |
| PA 34:1 | 673.5 | 153 | DAG 18:2/18:0 | 638.5 | 341.2 | TAG 54:3 | 902.8 | | |  |
| PA 36:6 | 691.5 | 153 | DAG 18:1/18:0 | 640.5 | 341.2 | TAG 54:2 | 904.8 | | |  |
| PA 36:5 | 693.5 | 153 | TAG 46:2 | 792.7 |  | TAG 54:0 | 908.8 | | |  |
| PA 36:4 | 695.5 | 153 | TAG 48:2 | 820.8 |  | TAG 56:8 | 920.9 | | |  |
| PA 36:3 | 697.5 | 153 | TAG 48:0 | 824.8 |  |  |  | | |  |
| PA 36:2 | 699.5 | 153 | TAG 50:4 | 844.8 |  |  |  | | |  |
| PA 36:1 | 701.5 | 153 | TAG 50:3 | 846.8 |  |  |  | | |  |

*MRM (multiple reaction monitoring) transitions for MGDG, DGDG, PA, and DAG species; as well as SIM (selective ion monitoring) transitions for TAG species, SIM transitions only have Q1.
