# Supplementary material for: GDSL lipases modulate immunity through lipid homeostasis in rice
Source: PLoS Pathog. 2017 Nov 13;13(11):e1006724. doi: 10.1371/journal.ppat.1006724 (PMC5703576; doi:10.1371/journal.ppat.1006724)
Supplement: S1 Fig — (A) Gene structure of OsGLIP1 and OsGLIP2. (B) A phylogenetic tree of GDSL lipases in rice. Protein sequences were aligned with CLUSTAL W, and the phylogenetic tree was constructed with MEGA4. (PDF) [file ppat.1006724.s004.pdf]

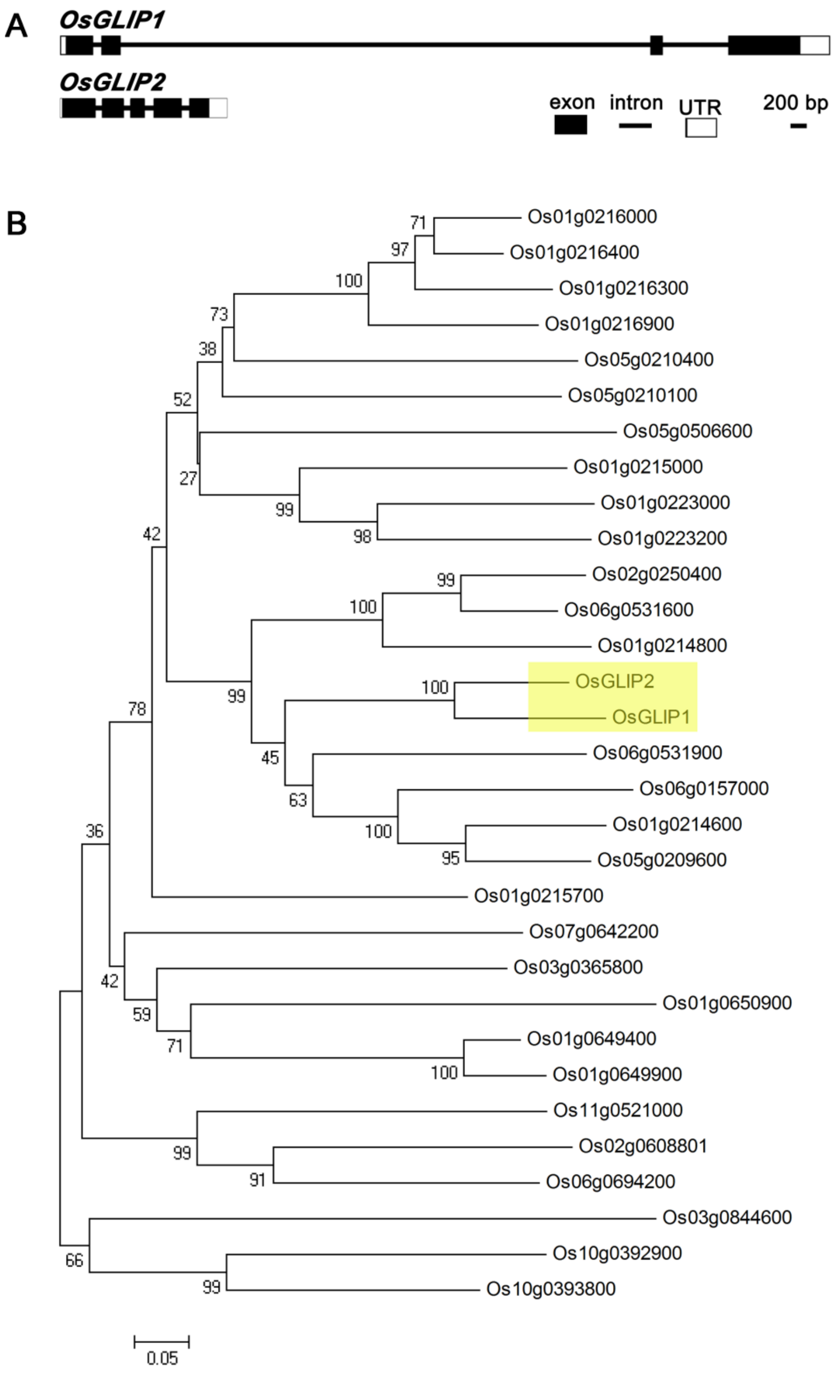

S1 Fig. Phylogenetic analysis of rice GDSL lipases

(A) Gene structure of OsGLIP1 and OsGLIP2. (B) A phylogenetic tree of GDSL lipases in rice. Protein sequences were aligned with CLUSTAL W, and the phylogenetic tree was constructed with MEGA4.
