# Supplementary material for: GDSL lipases modulate immunity through lipid homeostasis in rice
Source: PLoS Pathog. 2017 Nov 13;13(11):e1006724. doi: 10.1371/journal.ppat.1006724 (PMC5703576; doi:10.1371/journal.ppat.1006724)
Supplement: S2 Fig — The expression of OsGLIP1 and OsGLIP2 in 9 independent OsGLIP1-OE (A) and OsGLIP2-OE (B) lines and 8 independent OsGLIP1/2-RNAi lines (C) was compared to the wild-type control with normalization to rice Actin1 gene. Error bars, ± SD (n = 3). (PDF) [file ppat.1006724.s005.pdf]

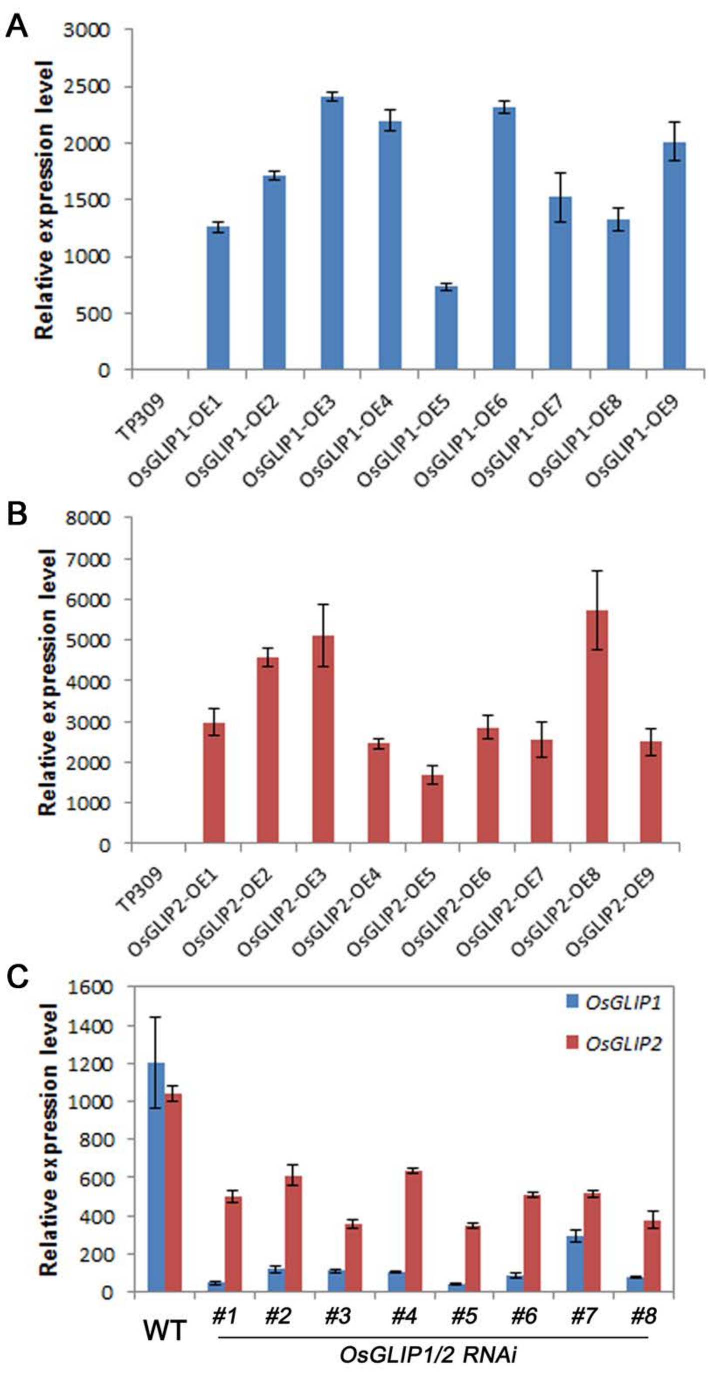

S2 Fig. Relative expression levels of *OsGLIP1* and *OsGLIP2* in overexpression and RNAi plants

The expression of *OsGLIP1* and *OsGLIP2* in 9 independent *OsGLIP1-OE* (A) and *OsGLIP2-OE* (B) lines and 8 independent *OsGLIP1/2*-RNAilines (C) was compared to the wild-type control with normalization to rice Actin1 gene. Error bars, ± SD (n = 3).
