# Supplementary material for: GDSL lipases modulate immunity through lipid homeostasis in rice
Source: PLoS Pathog. 2017 Nov 13;13(11):e1006724. doi: 10.1371/journal.ppat.1006724 (PMC5703576; doi:10.1371/journal.ppat.1006724)
Supplement: S3 Fig — The lipids, PC (A), PI (B), PE (C) and PG (D), are presented as the number of carbon atoms: total double bonds in the fatty acyl groups. Data are shown as means ± SD (n = 5) of mixed leaf samples from three representative transgenic lines. *P < 0.05 or **P < 0.01, by Student’s t-test and Bonferroni correction for multiple (three comparisons) tests. (PDF) [file ppat.1006724.s006.pdf]

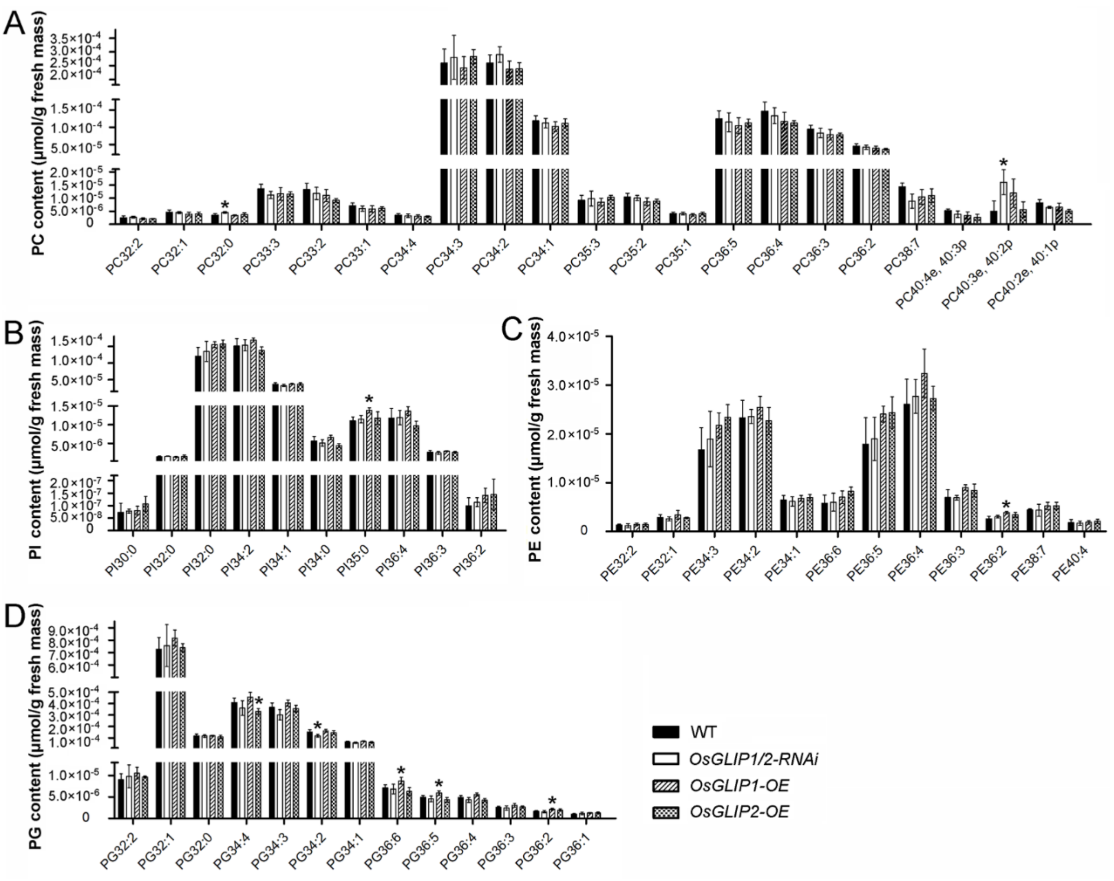

S3 Fig. Profiling of individual molecular species of lipids

Lipids, PC (A), PI (B), PE (C) and PG (D), are presented as the number of carbon atoms: total double bonds in the fatty acyl groups. Data are shown as means ± SD (n = 5) of mixed leaf samples from three representative transgenic lines. *P < 0.05 or **P < 0.01, by Student's t-test and Bonferroni correction for multiple (three comparisons) tests.
