# Supplementary material for: GDSL lipases modulate immunity through lipid homeostasis in rice
Source: PLoS Pathog. 2017 Nov 13;13(11):e1006724. doi: 10.1371/journal.ppat.1006724 (PMC5703576; doi:10.1371/journal.ppat.1006724)
Supplement: S4 Fig — (A) Lesion lengths in the leaves of wild-type and independent OsGLIP1-GFP and OsGLIP2-GFP transgenic plants. Plants at booting stage were infected with Xoo. Lesion length was calculated at 14 dpi. Data are shown as means ± SD (n > 10). Student’s t-test, **P < 0.01. (B) Western blot analysis with GFP antibodies confirmed the accumulation of the fusion proteins of OsGLIP1-GFP and OsGLIP2-GFP in the transgenic plants. Rubisco staining was used as loading control. (C) Bacterial growth during 8 days of inoculation in the representative transgenic lines. Data are shown as means ± SD (n = 3). Asterisks indicate significant difference in comparison with the wild-type control (Student’s t-test, *P < 0.05; ** P < 0.01). (PDF) [file ppat.1006724.s007.pdf]

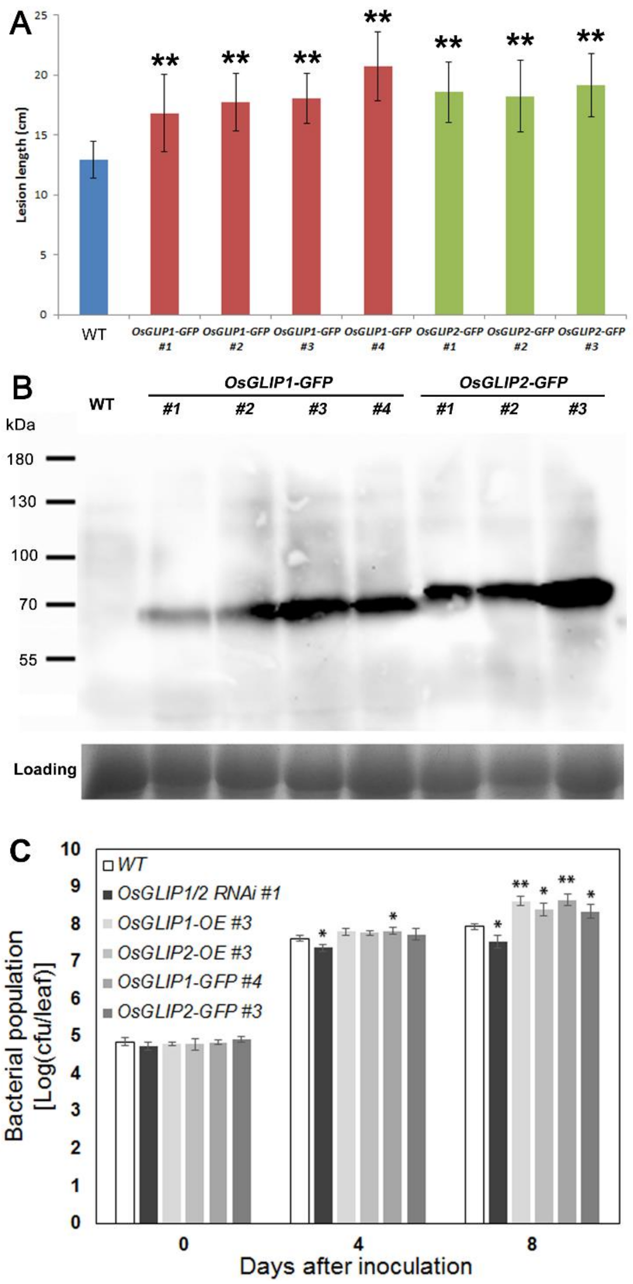

S4 Fig. Enhanced disease susceptibility in OsGLIP1-GFP and OsGLIP2-GFP transgenic plants

(A) Lesion lengths in the leaves of wild-type and independent *OsGLIP1-GFP* and *OsGLIP2-GFP* transgenic plants. Plants at booting stage were infected with *Xoo*. Lesion length was calculated at 14 dpi. Data are shown as means ± SD (n > 10). Student's *t*-test, **$P < 0.01$. (B) Western blot analysis with GFP antibodies confirmed the accumulation of the fusion proteins of OsGLIP1-GFP and OsGLIP2-GFP in the transgenic plants. Rubisco staining was used as loading control. (C) Bacterial growth during 8 days of inoculation in the representative transgenic lines. Data are shown as means ± SD ($n = 3$). Asterisks indicate significant difference in comparison with the wild-type control (Student's *t*-test, *$P < 0.05$; ** $P < 0.01$).
