# Supplementary material for: GDSL lipases modulate immunity through lipid homeostasis in rice
Source: PLoS Pathog. 2017 Nov 13;13(11):e1006724. doi: 10.1371/journal.ppat.1006724 (PMC5703576; doi:10.1371/journal.ppat.1006724)
Supplement: S5 Fig — The root tips of transgenic plants were incubated in 25% sucrose to induce cell plasmolysis and then observed under confocal microscopy. (A, B) Confocal images of OsGLIP1–GFP (A) and OsGLIP2–GFP (B) after cell plasmolysis. Scale bars = 20 μm. (C) OsGLIP1-GFP root cells without plasmolysis to show the endomembrane systems that link OsGLIP1-GFP labelled vesicles. Scale bar = 20 μm. (PDF) [file ppat.1006724.s008.pdf]

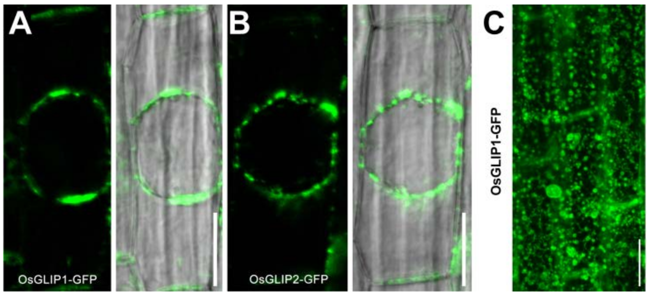

S5 Fig. Plasmolysis analysis of *OsGLIP1-GFP* and *OsGLIP2-GFP* transgenic plants cells

The root tips of transgenic plants were incubated in 25% sucrose to induce cell plasmolysis and then observed under confocal microscopy. (A, B) Confocal images of *OsGLIP1–GFP* (A) and *OsGLIP2–GFP* (B) after cell plasmolysis. Scale bars = 20 μm. (C) *OsGLIP1-GFP* root cells without plasmolysis to show the endomembrane systems that link OsGLIP1-GFP labelled vesicles. Scale bar = 20 μm.
