# Supplementary material for: GDSL lipases modulate immunity through lipid homeostasis in rice
Source: PLoS Pathog. 2017 Nov 13;13(11):e1006724. doi: 10.1371/journal.ppat.1006724 (PMC5703576; doi:10.1371/journal.ppat.1006724)
Supplement: S6 Fig — Protoplasts were generated from seedlings of transgenic plants expressing OsGLIP1-GFP and OsGLIP2-GFP fusion protein. Note the overlapping of GFP green signals and Nile Red stained red signals, while OsGLIP1-GFP and OsGLIP2-GFP were not associated with chloroplasts. Scale bars = 10 μm. (PDF) [file ppat.1006724.s009.pdf]

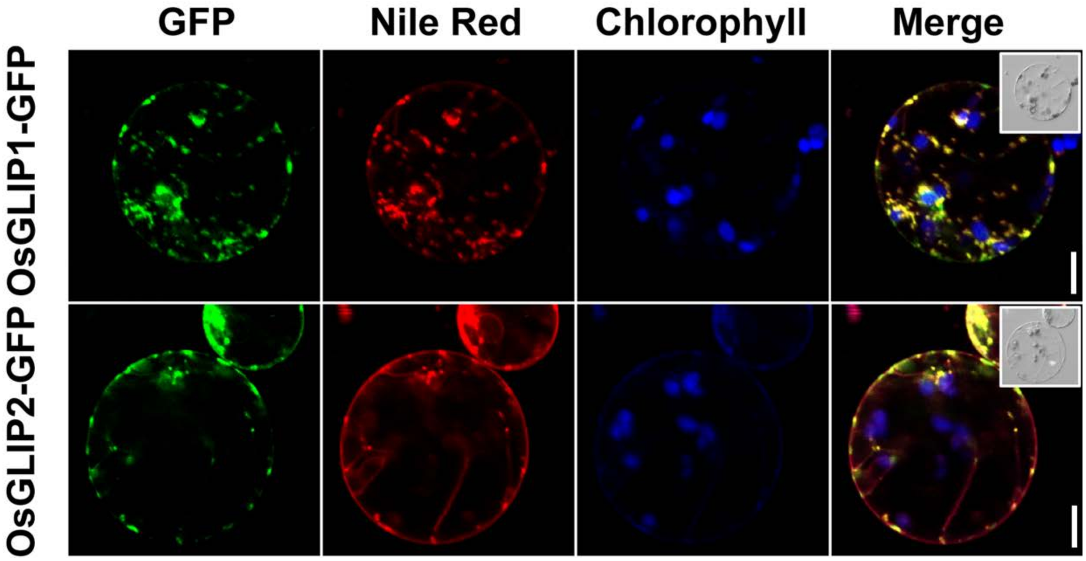

S6 Fig. Co-localization of OsGLIP1 and OsGLIP2 with Nile Red in rice protoplasts

Protoplasts were generated from seedlings of transgenic plants expressing OsGLIP1-GFP and OsGLIP2-GFP fusion protein. Note the overlapping of GFP green signals and Nile Red stained red signals, while OsGLIP1-GFP and OsGLIP2-GFP were not associated with chloroplasts. Scale bars = 10 μm.
