# Supplementary material for: GDSL lipases modulate immunity through lipid homeostasis in rice
Source: PLoS Pathog. 2017 Nov 13;13(11):e1006724. doi: 10.1371/journal.ppat.1006724 (PMC5703576; doi:10.1371/journal.ppat.1006724)
Supplement: S7 Fig — Two-week-old rice leaves were fed with 100μM MGDG (A) and DGDG (B) for 24 hours and subsequently used for lipid extraction and measurements. Data are shown as means ± SD of four biological replicates. Student’s t-test. *P < 0.05. (PDF) [file ppat.1006724.s010.pdf]

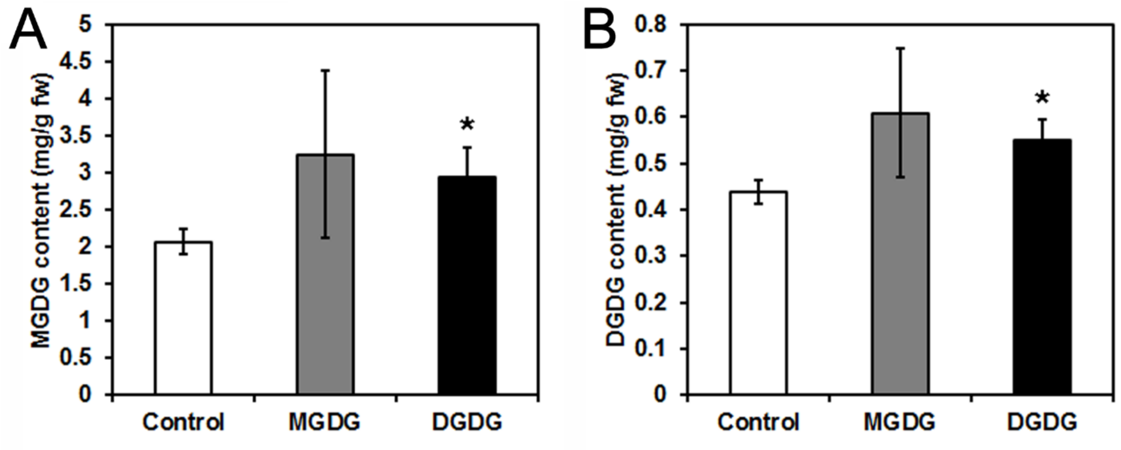

S7 Fig. The contents of MGDG and DGDG in fed leaves

Two-week-old rice leaves were fed with 100μM MGDG and DGDG for 24 hours and subsequently used for lipid extraction and measurements. Data are shown as means ± SD of four biological replicates. Student's *t*-test. *$P < 0.05$.
